# C_18_H_17_NO_6_ and Its Combination with Scutellarin Suppress the Proliferation and Induce the Apoptosis of Human Glioma Cells via Upregulation of Fas-Associated Factor 1 Expression

**DOI:** 10.1155/2019/6821219

**Published:** 2019-02-20

**Authors:** Xiu-Ying He, Liu-Lin Xiong, Qing-Jie Xia, Yang-Yang Wang, Xiao-Ming Zhao, Ruo-Lan Du, Jin Huang, Xiao-Qiong He, Jia-Liu, Ting-Hua Wang

**Affiliations:** ^1^Institute of Neurological Disease, Department of Anesthesiology, Translational Neuroscience Center, West China Hospital, Sichuan University, Chengdu, China; ^2^Department of Anesthesiology, Traditional Chinese Medicine Hospital of Southwest Medical University, Luzhou, China; ^3^Department of Histology, Embryology and Neurobiology, West China School of Preclinical and Forensic Medicine, Sichuan University, Chengdu, China; ^4^Institute of Neuroscience, Laboratory Zoology Department, Kunming Medical University, Kunming, China; ^5^School of Public Health, Kunming Medical University, Kunming, China

## Abstract

**Background:**

Glioma is the most common malignant brain tumor and the patients are prone to poor prognosis. Due to limited treatments, new drug exploration has become a general trend. Therefore, the objective of this study is to investigate the effect of the new drugs C_18_H_17_NO_6_ and its combination with Scutellarin on glioma cells and the underlying mechanism.

**Method:**

U251 and LN229 cells were administrated with C_18_H_17_NO_6_ and its combination with Scutellarin. The proliferation ability of glioma cells was determined by cell counting kit-8, plate clone formation assay, and EdU incorporation assay. The cell cycle and apoptosis detection were detected by flow cytometry. Moreover, TUNEL assay was also used for cell apoptosis analysis. Then, the transfer ability of cells was achieved through wound healing assay. Furthermore, polymerase chain reaction (PCR) test and western bolt analysis were used to detect the mRNA expression and protein expression, respectively. Lastly, immunofluorescence was for the purity identification of astrocyte.

**Result:**

The results showed that, with the increasing dose of C_18_H_17_NO_6_, the cell inhibition rate, the cells in G1 phase, and the apoptosis rate were gradually increased, but the clone number, proliferation rate, and the cells in G2 and S phases were gradually decreased in comparison with control group. However, with the increase of C_18_H_17_NO_6_, the transferred rate of U251 and LN229 was not significantly augmented, expect that on U251 in C_18_H_17_NO_6_ 5 *μ*M group. In addition, Scutellarin 200 *μ*M has little effect on proliferation, with the inhibition rate 10-20% and proliferation rate except U251 in Scutellarin 200 *μ*M group similar to that in control group. Moreover, compared to control group, Scutellarin 300 *μ*M increased the U251 cells in G2 and S phases and the apoptosis rate of LN229 but decreased the LN229 cells in G2 and S phases. Besides, in Scutellarin 200 *μ*M group, the transfer ability of LN229 was inhibited, but not in U251. Furthermore, if C_18_H_17_NO_6_ was combined with Scutellarin 200/300*μ*M, the proliferation and transferred ability were suppressed and the apoptosis was elevated in LN229 cell in comparison with C_18_H_17_NO_6_ alone. Dramatically, the combined effect on U251 was the exact opposite. Importantly, there was little toxicity on astrocyte under the dose of C_18_H_17_NO_6_ and Scutellarin in the study. In molecular level, the mRNA and protein expression of Fas-associated factor 1 (FAF1) expression in U251 and LN229 were upregulated by C_18_H_17_NO_6_ and its combination with Scutellarin, especially the protein expression.

**Conclusion:**

C_18_H_17_NO_6_ could efficiently suppress cell proliferation and induce cell apoptosis in glioma cells, and its combination with Scutellarin had a promoting effect, in which the underlying mechanism referred to the upregulation of Fas-associated factor 1.

## 1. Introduction

The Benign and malignant central nervous system neoplasms are usually derived from glial cells (i.e., astrocytes, oligodendrocytes, and ependymocytes) [1, 2]. Gliomas account for approximately 80% of all malignant primary central nervous system tumors [3–5]. As glioma is from the neoplastic glial cells, or neuroglia, it is further classified by the World Health Organization (WHO) as astrocytoma, oligodendroglioma, mixed oligoastrocytoma, and ependymoma [6, 7]. Additional stratification of tumor grade is determined by tumor histopathological impression, specifically by the presence of nuclear pleomorphism, increased mitotic activity and cellularity, endothelial cell proliferation, and necrosis (with all four features-usually necrosis is the fourth feature-indicating a grade IV tumor) [8, 9]. The histological subtype and grade are not only associated with malignant potential, but also associated with response to treatment and survival [10, 11]. Stage I and II gliomas are lower-risk tumors and have a better prognosis, whereas stage III and IV gliomas, including anaplastic astrocytomas and glioblastomas, are high-grade malignant tumors [12, 13]. Nowadays, although progress has been made in treatment such as surgery, chemotherapy, and radiotherapy, recurrence after standard therapies is inevitable, and the median survival of patients with high-grade malignant gliomas is no more than 14 months [14, 15]. The 5-year survival rate of patients with glioblastoma is less than 3% [16]. Thus, finding susceptible cells and molecules on which new therapeutic options are achieved has been the focus of diagnosis and treatment of gliomas. In particular, the development of new drugs with strong anticancer effect but no toxicity has been a must.

FAS-associated factor 1, FAF1, is an evolutionarily conserved protein with several protein interaction domains including ubiquitin-associating (UBA), ubiquitin like 1 and 2 (UBL1; UBL2), and ubiquitin regulatory X (UBX) domains [17, 18]. These domains can bind to FAS antigen and initiate the apoptosis progress or enhance apoptosis [19]. Most of all, FAF1 can serve as a tumor suppressor involving in the regulation of apoptosis and NF-kappaB activity in HeLa cells [20]. On the one hand, FAF1 was downregulated in gastric carcinoma, which activated the NF-*κ*B signaling to promote proliferation, infiltration, and lymph node metastasis of gastric carcinoma [21]. On the other hand, it had been reported that the expression of Fas/Apo-1 was inversely correlated with bcl-2 and seemed to be a good prognostic factor which may reflect the relative homeostasis of apoptotic pathway [22]. However, when the induced apoptosis drugs C_18_H_17_NO_6_ and their combination with Scutellarin were administrated, the expression of FAF1 and its function in glioma have been unknown.

Therefore, the purpose of this study is to confirm that the new drugs, C_18_H_17_NO_6_ and its combination with Scutellarin, can suppress the proliferation and malignant transformation of glioma cells. Additionally, we determine whether Fas-associated factor 1 has been involved in the underlying molecular mechanism.

## 2. Results

### 2.1. IC50 of C_18_H_17_NO_6_ and Scutellarin in Glioma Cell Lines

**Figure 1:**
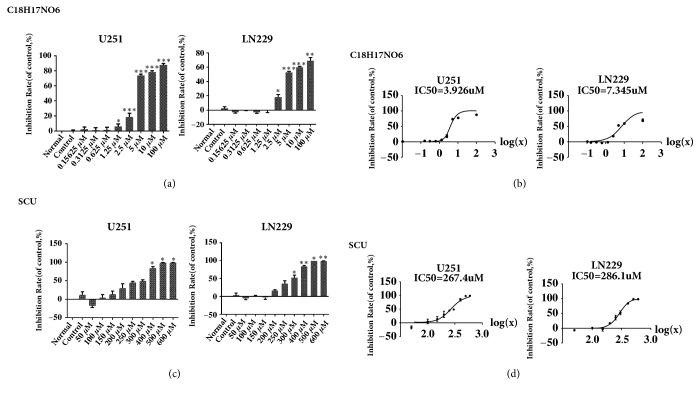
IC50 of C_18_H_17_NO_6_ and Scutellarin in glioma cell lines. (a) Inhibition rate on U251 and LN229 cells treated with different concentration of C_18_H_17_NO_6_. (b) IC50 curve and IC50 of C_18_H_17_NO_6_ on U251 and LN229 cells. (c) Inhibition rate on U251 and LN229 cells treated with different concentration of Scutellarin (SCU). (d) IC50 curves and IC50 of Scutellarin on U251 and LN229. Data are shown as mean + SD (n=3). Normal group, the normally cultured cell, with no intervention including the solvent. *∗* versus control (DMSO), *∗* P < 0.05, *∗∗* P < 0.01, and *∗∗∗* P < 0.001.

After 48 hours of C_18_H_17_NO_6_ intervention, we found that C_18_H_17_NO_6_ could effectively inhibit the proliferation of U251 and LN229 in a dose-dependent manner, and its IC50 (the concentration of C_18_H_17_NO_6_ when the inhibition rate reached 50%) on U251 and LN229 were 3.926 *μ*M and 7.345 *μ*M, respectively, and the 95% confidence intervals (CI) were 3.482 *μ*M to 4.427 *μ*M and 5.266 *μ*M to 10.24 *μ*M, respectively (Figures 1(a) and 1(b) and Data S1. a).

Similarly, after 48h intervention, Scutellarin also inhibited the proliferation of U251 and LN229 in a dose-dependent manner. The IC50 on U251 and LN229 were 267.4 *μ*M and 286.1 *μ*M, and the 95% CI were 250.4 *μ*M to 285.7 *μ*M and 277.7 *μ*M to 294.9 *μ*M, respectively (Figures 1(c) and 1(d) and Data S1. b).

### 2.2. Effect of C_18_H_17_NO_6_ and Its Combination with Scutellarin on the Cell Viability of Glioma Cells

**Figure 2:**
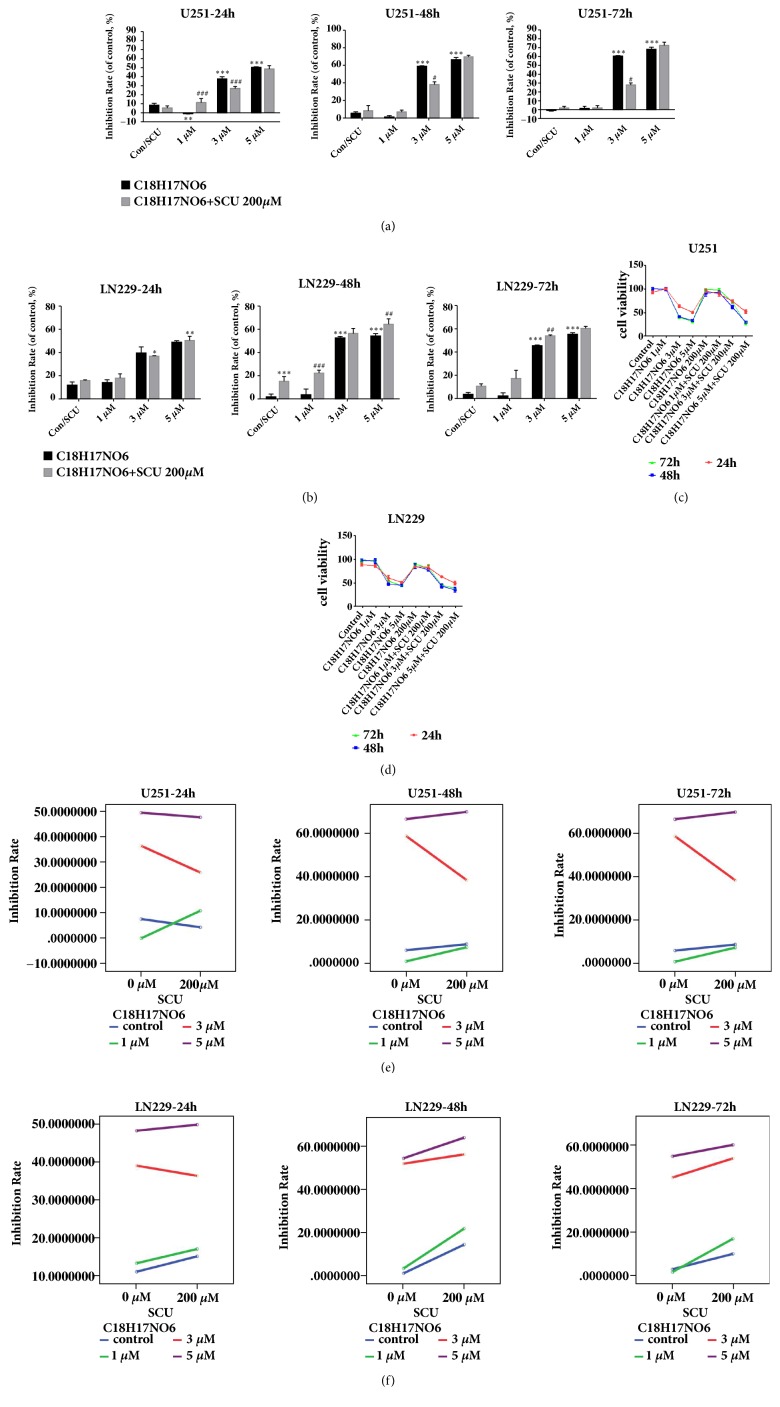
Effect of C_18_H_17_NO_6_ and its combination with Scutellarin on the cell viability of glioma cells. (a, b) Inhibition rate on U251 and LN229 cells at 24, 48, and 72 h after intervention of different concentration of C_18_H_17_NO_6_ and its combination with Scutellarin (SCU) 200 *μ*M. (c, d) Cell viability of U251 and LN229 cells at 24, 48, and 72 h after intervention of different concentrations of C_18_H_17_NO_6_ and its combination with Scutellarin 200 *μ*M. The effect peaked at 48 h after dosing. (e, f) The interaction plots of inhibition rate of C_18_H_17_NO_6_ and its combination with Scutellarin 200 *μ*M on U251 and LN229. The P values in each graph were 0.000, 0.000, 0.000, 0.45, 0.000, and 0.000 in turn. *∗* versus control (DMSO), # C_18_H_17_NO_6_ x versus C_18_H_17_NO_6_ x + SCU 200 *μ*M, *∗*/# P < 0.05, *∗∗*/## P < 0.01, and *∗∗∗*/### P < 0.001.

The cell viability of U251 and LN229 after 24, 48, and 72 h intervened by C_18_H_17_NO_6_ and its combination with Scutellarin 200 *μ*M was measured by the CCK8 kit. C_18_H_17_NO_6_ was found to inhibit the proliferation of U251 and LN229 in a concentration-dependent manner (Figures 2(a), 2(b), 2(c), and 2(d) and Data S2. a, b, c). Compared with control group, although the inhibition rate on U251 and LN229 in C_18_H_17_NO_6_ 1 *μ*M group did not differ at any of the three time points, the inhibition rate on U251 and LN229 was increased in C_18_H_17_NO_6_ 3 *μ*M group and C_18_H_17_NO_6_ 5 *μ*M group. Moreover, except that at 24h the inhibition rate on LN229 was not statistically different from that of control group (P>0.05), other differences were statistically significant (P<0.001) (Figures 2(a) and 2(b)). In addition, Scutellarin 200 *μ*M showed no inhibitory effect on U251 (P>0.05), but the inhibition rate on LN229 was increased compared with control group, and the difference was statistically significant only at 48h (P<0.001) (Figures 2(a) and 2(b)). When C_18_H_17_NO_6_ was combined with Scutellarin 200 *μ*M, the inhibition rate of U251 was decreased (C_18_H_17_NO_6_ 3 *μ*M versus C_18_H_17_NO_6_ 3 *μ*M plus SCU 200 *μ*M at 24 h, 48 h, and 72 h P<0.05). However, at 24 h, compared with C_18_H_17_NO_6_ 1 *μ*M, C_18_H_17_NO_6_ 1 *μ*M plus SCU 200 *μ*M increased the inhibition rate (P<0.001) (Figure 2(a)). On the other hand, when combined with Scutellarin 200 *μ*M, the inhibition rate on LN229 was higher than that of C_18_H_17_NO_6_ alone (Figure 2(b)). The results showed that Scutellarin 200 *μ*M can promote the inhibition of C_18_H_17_NO_6_ on LN229 and antagonize the inhibitory effect on U251. At the same time, there was an interactive effect between the two drugs (Figures 2(e) and 2(f)). Moreover, the inhibition rate on U251 and LN229 by C_18_H_17_NO_6_ and its combination with Scutellarin 200 *μ*M were also time-dependent, and its inhibitory effect increased with time going, but the inhibition rate peaked at 48 h after dosing (Figures 2(c) and 2(d)).

### 2.3. Effects of C_18_H_17_NO_6_ and Its Combination with Scutellarin on the Clone Formation of Glioma Cells

**Figure 3:**
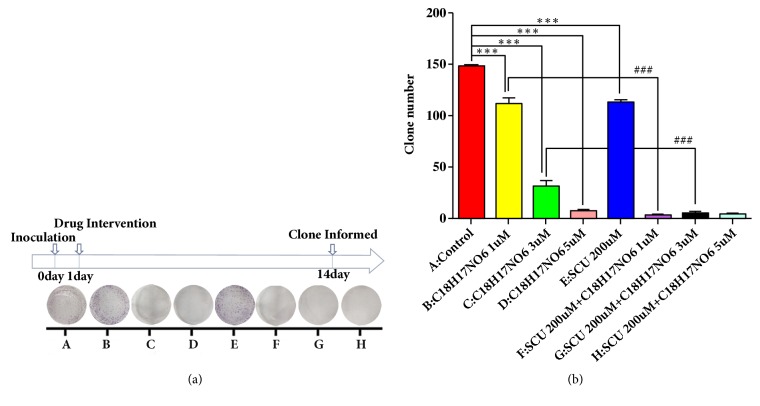
Effects of C_18_H_17_NO_6_ and its combination with Scutellarin on the clone formation of glioma cells. (a) The pictures show the effect of C_18_H_17_NO_6_ and its combination with Scutellarin (SCU) 200 *μ*M on the cloning of LN229 cells. (b) Quantification of the clone number of LN229 cells with C_18_H_17_NO_6_ and its combination with Scutellarin 200 *μ*M. *∗* versus control (DMSO), # C_18_H_17_NO_6_ x versus C_18_H_17_NO_6_ x + SCU 200 *μ*M, *∗*/# P < 0.05, *∗∗*/## P <0.01, and *∗∗∗*/### P < 0.001. One clone has more than 50 cells.

It was found that, as the concentration of C_18_H_17_NO_6_ increased, the clone number of LN229 gradually decreased, which is significantly lower than that of control group (P<0.001) (Figures 3(a) and 3(b) and Data S3). Additionally, the clone number in the Scutellarin 200 *μ*M group also decreased compared with control group, and the difference was statistically significant (P<0.001) (Figures 3(a) and 3(b) and Data S3). Moreover, when combined with Scutellarin 200 *μ*M on the basis of C_18_H_17_NO_6_, Scutellarin 200 *μ*M further reduced the formation of LN229 clones, and the differences about the comparison between C_18_H_17_NO_6_ 1 *μ*M plus SCU 200 *μ*M group and C_18_H_17_NO_6_ 1 *μ*M group and between C_18_H_17_NO_6_ 3 *μ*M plus SCU 200 *μ*M group and C_18_H_17_NO_6_ 3 *μ*M group were statistically significant (P<0.001) (Figures 3(a) and 3(b) and Data S3).

### 2.4. Effect of C_18_H_17_NO_6_ and Its Combination with Scutellarin on Proliferation of Glioma Cells

**Figure 4:**
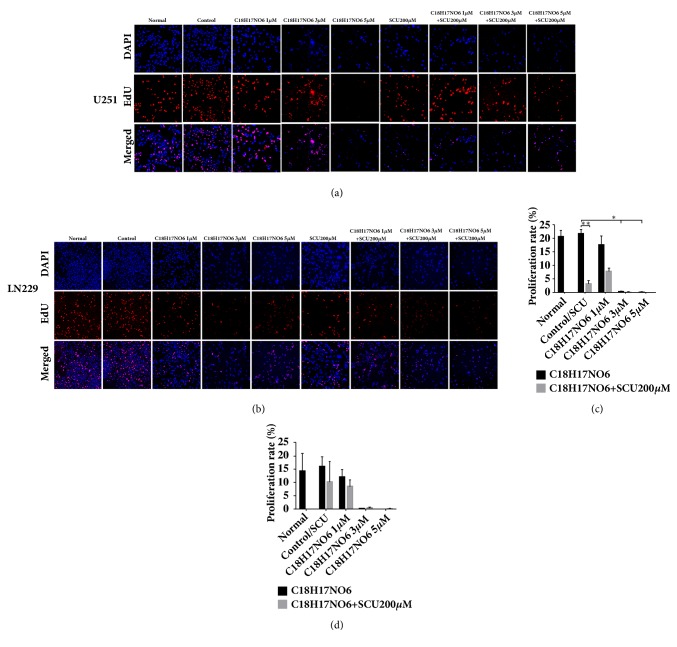
Effect of C_18_H_17_NO_6_ and its combination with Scutellarin on proliferation of glioma cells. (a, b) Fluorescence pictures show the effect of C_18_H_17_NO_6_ and its combination with Scutellarin (SCU) 200 *μ*M on the proliferation of U251 and LN229 at 48h of their intervention. (c, d) The proliferation rate of U251 and LN229 at 48h of C_18_H_17_NO_6_ and its combination with Scutellarin 200 *μ*M intervention. *∗* versus control (DMSO), # C_18_H_17_NO_6_ x versus C_18_H_17_NO_6_ x + SCU 200*μ*M, *∗*/# P < 0.05, *∗∗*/## P < 0.01, and *∗∗∗*/### P < 0.001.

EdU can replace thymine (T) in the replication of DNA during the cell proliferation period. By detecting the intensity of the fluorescent dye bound to EdU, it can quickly estimate the DNA replication activity and further accurately determine the cell proliferation ability. Compared with control group, the proliferation rate of U251 in C_18_H_17_NO_6_ 1 *μ*M group was not significantly different (P>0.05), but the proliferation rates of U251 in C_18_H_17_NO_6_ 3 *μ*M group and C_18_H_17_NO_6_ 5 *μ*M group were significantly decreased (P<0.05) (Figures 4(a) and 4(c) and Data S4. a, c). In addition, Scutellarin 200 *μ*M significantly decreased the proliferation rate of U251 (P<0.01) (Figures 4(a) and 4(c) and Data S4. a, c). Moreover, combination with Scutellarin 200 *μ*M can further inhibit the DNA replication ability of U251 compared with C_18_H_17_NO_6_ alone, but the difference was not significant (P > 0.05) (Figures 4(a) and 4(c) and Data S4. a, c).

For LN229 cell, compared with control group, the proliferation rate of LN229 decreased gradually with the increase of the concentration of C_18_H_17_NO_6_ (P>0.05) (Figures 4(b) and 4(d) and Data S4. b, c). At the same time, when administrated with Scutellarin 200 *μ*M, the proliferation rate of LN229 also decreased, but there was no significant difference compared with control group (P>0.05) (Figures 4(b) and 4(d) and Data S4. b, c). Additionally, compared with C_18_H_17_NO_6_ alone, the proliferation rate of LN229 was further reduced when combined with Scutellarin 200 *μ*M, but the difference was not significant (P>0.05) (Figures 4(b) and 4(d) and Data S4. b, c).

### 2.5. Effect of C_18_H_17_NO_6_ and Its Combination with Scutellarin on Cell Cycle of Glioma Cells by Flow Cytometry Analysis

**Figure 5:**
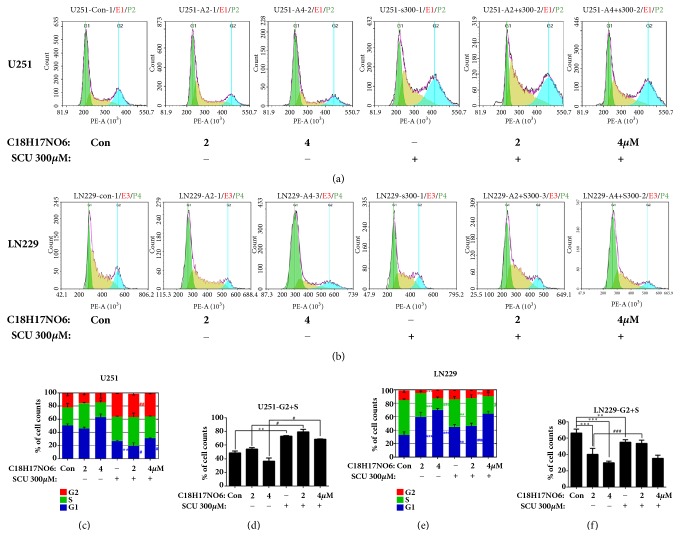
Effect of C_18_H_17_NO_6_ and its combination with Scutellarin on cell cycle of glioma cells by flow cytometry analysis. (a, b) Cell cycle distribution diagrams show the proportion of U251 and LN229 in G1, S, and G2 phases intervened by C_18_H_17_NO_6_ and its combination with Scutellarin (SCU) 300 *μ*M. (c, e) The proportion of U251 and LN229 cells in the G1, S, and G2 phases intervened by C_18_H_17_NO_6_ and its combination with Scutellarin 300 *μ*M. (d, f) The sum proportion of U251 and LN229 cells in the S and G2 phases intervened by C_18_H_17_NO_6_ and its combination with Scutellarin 300 *μ*M. *∗* versus control (DMSO), # C_18_H_17_NO_6_ x versus C_18_H_17_NO_6_ x + SCU 300 *μ*M, *∗*/# P < 0.05, *∗∗*/## P < 0.01, and *∗∗∗*/### P < 0.001.

The cell cycle of the glioma cells was detected by flow cytometry. After 48 hours of intervention, we found that, with the increasing dose of C_18_H_17_NO_6_, the proportion of U251 cells in G1 phase gradually increased, and the proportion of U251 cells in G2 and S phase gradually decreased, but the difference was all not significant (P > 0.05) in comparison with control group (Figures 5(a), 5(c), and 5(d) and Data S5). However, compared with control group, the proportion of U251 cells in the G2 and S phases of the Scutellarin 300 *μ*M group increased (P < 0.05), while the proportion of U251 cells in the G1 phase decreased (P < 0.05) (Figures 5(a), 5(c), and 5(d) and Data S5). Besides, when Scutellarin 300 *μ*M was combined with C_18_H_17_NO_6_, the proportion of U251 cells in G2 and S phases increased (P < 0.05), while U251 cells in G1 phase decreased (P < 0.05) compared with the C_18_H_17_NO_6_ alone (Figures 5(a), 5(c), and 5(d) and Data S5).

Similarly, as the concentration of C_18_H_17_NO_6_ increased, the proportion of LN229 cells in the G1 phase gradually increased (P < 0.001) after 48 hours of intervention compared to control group, while the proportion of LN229 cells in the G2 and S phases gradually decreased (P < 0.001) (Figures 5(b), 5(e), and 5(f) and Data S5). Compared with control group, the proportion of LN229 cells in the G2 and S phases of Scutellarin 300 *μ*M group also decreased (P < 0.01), while the proportion of LN229 cells in the G1 phase increased (P < 0.01) (Figures 5(b), 5(e), and 5(f) and Data S5). In addition, the proportion of LN229 cells in the G2 and S phases increased in C_18_H_17_NO_6_ plus Scutellarin 300 *μ*M compared to C_18_H_17_NO_6_ alone, while the LN229 cells in the G1 phase decreased. Moreover, the differences in the proportion of LN229 cells at G2 and S phase and at G1 phase were statistically significant between C_18_H_17_NO_6_ 2 *μ*M group and C_18_H_17_NO_6_ 2*μ*M plus Scutellarin 300 *μ*M group (P < 0.05) (Figures 5(b), 5(e), and 5(f) and Data S5).

### 2.6. Effect of C_18_H_17_NO_6_ and Its Combination with Scutellarin on Apoptosis of Glioma Cells by TUNEL Assay

**Figure 6:**
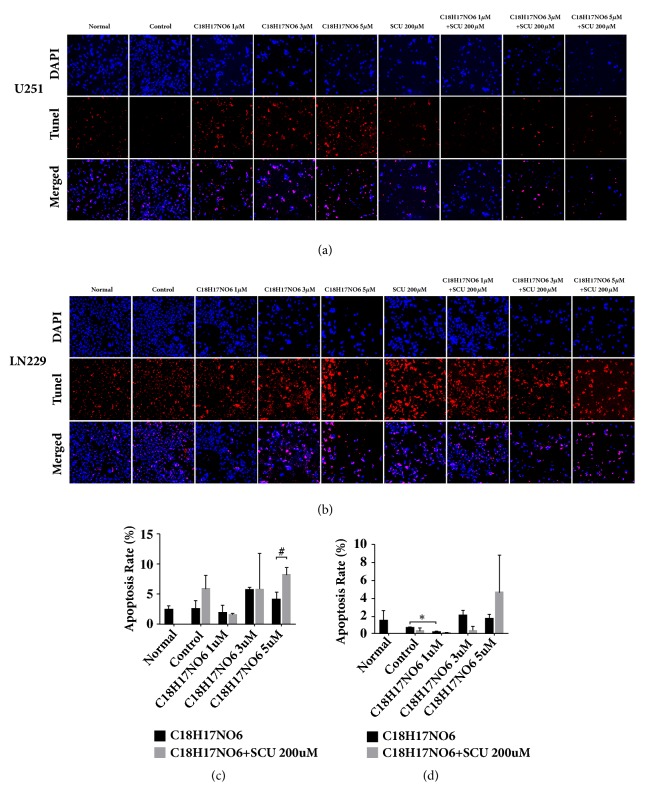
Effect of C_18_H_17_NO_6_ and its combination with Scutellarin on apoptosis of glioma cells by TUNEL assay. (a, b) Fluorescence pictures show the apoptosis of U251 and LN229 cells intervened by C_18_H_17_NO_6_ and its combination with Scutellarin (SCU) 200 *μ*M for 48h. (c, d) Quantifying the apoptosis rate of U251 and LN229 cells intervened by C_18_H_17_NO_6_ and its combination with Scutellarin 200 *μ*M for 48h. *∗* versus control (DMSO), # C_18_H_17_NO_6_ x versus C_18_H_17_NO_6_ x + SCU 200*μ*M, *∗*/# P < 0.05, *∗∗*/## P < 0.01, and *∗∗∗*/### P < 0.001.

The TUNEL assay was used to detect the apoptosis of glioma cells. The apoptosis rate of U251 and LN229 increased after 48 hours of intervention by C_18_H_17_NO_6_, but the apoptosis rate of U251 was not statistically significant compared with that in control group (P > 0.05) (Figures 6(a) and 6(c) and Data S6. a, c). For LN229, the apoptosis rate was significantly different between C_18_H_17_NO_6_ 1 *μ*M group and control group (P < 0.05), and the apoptosis rate of LN229 was increased in C_18_H_17_NO_6_ 3 *μ*M group compared with C_18_H_17_NO_6_ 1 *μ*M group (P > 0.05) (Figures 6(b) and 6(d) and Data S6. b, c). Besides, compared with control group, Scutellarin 200 *μ*M increased the apoptosis rate of U251, but it was not statistically significant (P > 0.05) (Figures 6(a) and 6(c) and Data S6. a, c). When Scutellarin 200 *μ*M was combined with C_18_H_17_NO_6_ 5*μ*M, the apoptosis rate of U251 increased in comparison with Scutellarin 200 *μ*M alone, and the difference was statistically significant (Figures 6(a) and 6(c) and Data S6. a, c). Though the apoptosis rate of LN229 in C_18_H_17_NO_6_ 5 *μ*M plus Scutellarin 200 *μ*M group was larger that in C_18_H_17_NO_6_ 5 *μ*M group, the difference was not statistically significant (Figures 6(b) and 6(d) and Data S6. b, c).

### 2.7. Effect of C_18_H_17_NO_6_ and Its Combination with Scutellarin on the Apoptosis of Glioma Cells by Flow Cytometry Analysis

**Figure 7:**
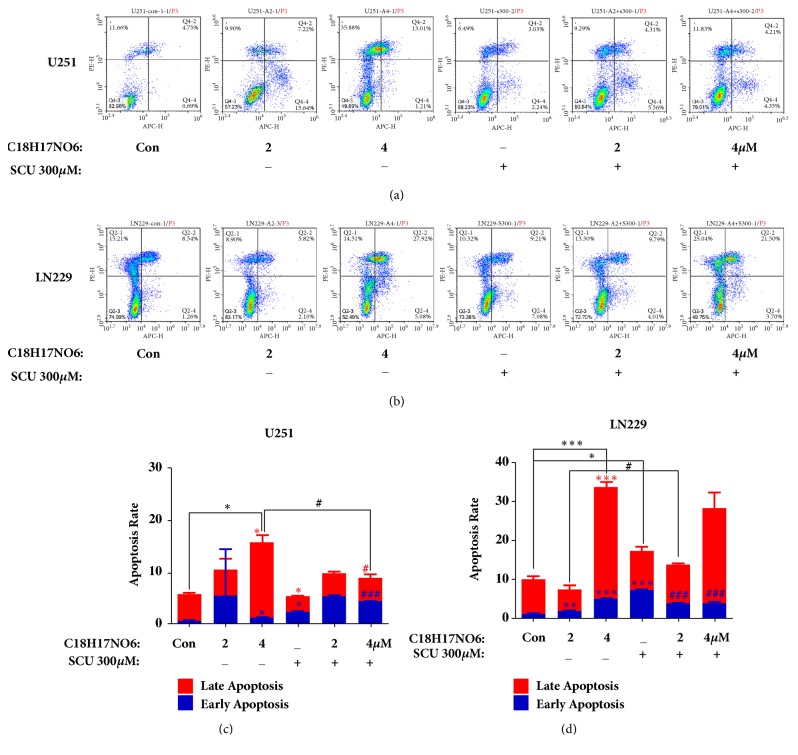
Effect of C_18_H_17_NO_6_ and its combination with Scutellarin on the apoptosis of glioma cells by flow cytometry analysis. (a, b) Apoptosis plots show the apoptosis of U251 and LN229 intervened by C_18_H_17_NO_6_ and its combination with Scutellarin (SCU) 300 *μ*M for 48h. (c, d) Quantify the early, late, and total apoptotic rates of U251 and LN229 cells intervened by C_18_H_17_NO_6_ and its combination with Scutellarin 300 *μ*M for 48h. *∗* versus control (DMSO), # C_18_H_17_NO_6_ x versus C_18_H_17_NO_6_ x + SCU 300 *μ*M, *∗*/# P < 0.05, *∗∗*/## P < 0.01, and *∗∗∗*/### P < 0.001.

Flow cytometry was also used to detect the apoptosis of glioma cells. After 48 hours of intervention with C_18_H_17_NO_6_, the early apoptosis rate, late apoptosis rate (necrosis), and total apoptosis rate of U251 were increased compared to control group, and all the differences between C_18_H_17_NO_6_ 4 *μ*M group and control group was significant(P < 0.05) (Figures 7(a) and 7(c) and Data S7). Then, compared with control group, Scutellarin 300 *μ*M increased the early apoptosis rate of U251 (P < 0.05) but decreased the late apoptosis rate of U251 (P < 0.05) and further there is no difference in the total apoptosis rate (Figures 7(a) and 7(c) and Data S7). When combined with Scutellarin 300 *μ*M, the total apoptosis rate of U251 did not increase. However, there were the higher early apoptotic rate (P < 0.001) and lower late apoptotic rate and total apoptotic rate (P < 0.05) in C_18_H_17_NO_6_ 4 *μ*M plus SCU 300 *μ*M group than that in C_18_H_17_NO_6_ 4 *μ*M group (Figures 7(a) and 7(c) and Data S7).

With regard to LN229 cell, after 48h intervention by C_18_H_17_NO_6_, the early apoptotic rate of LN229 increased, and the differences both C_18_H_17_NO_6_ 2 *μ*M group and C_18_H_17_NO_6_ 4*μ*M group were significantly higher than that of control group (P < 0.05) (Figures 7(b) and 7(d) and Data S7). Compared with control group, the late apoptosis rate and total apoptosis rate decreased in C_18_H_17_NO_6_ 2 *μ*M group (P > 0.05), but increased in C_18_H_17_NO_6_ 4 *μ*M group (P < 0.001) (Figures 7(b) and 7(d) and Data S7). Compared to control group, Scutellarin 300 *μ*M increased the early apoptosis rate and the total apoptosis rate of LN229 (P < 0.05), but the late apoptosis rate (Figures 7(b) and 7(d) and Data S7). Moreover, the early apoptosis rate (P < 0.001), late apoptosis rate (P > 0.05), and total apoptosis rate (P<0.05) were higher in C_18_H_17_NO_6_ 2 *μ*M plus SCU 300 *μ*M group than that in C_18_H_17_NO_6_ 2 *μ*M group (Figures 7(b) and 7(d) and Data S7). However, the early apoptotic rate (P < 0.001), late apoptotic rate (P > 0.05), and total apoptotic rate (P > 0.05) of C_18_H_17_NO_6_ 4 *μ*M plus SCU 300 *μ*M group decreased in comparison with C_18_H_17_NO_6_ 4 *μ*M group (Figures 7(b) and 7(d) and Data S7).

### 2.8. Effects of C_18_H_17_NO_6_ and Its Combination with Scutellarin on the Transferred Rate of Glioma Cells

**Figure 8:**
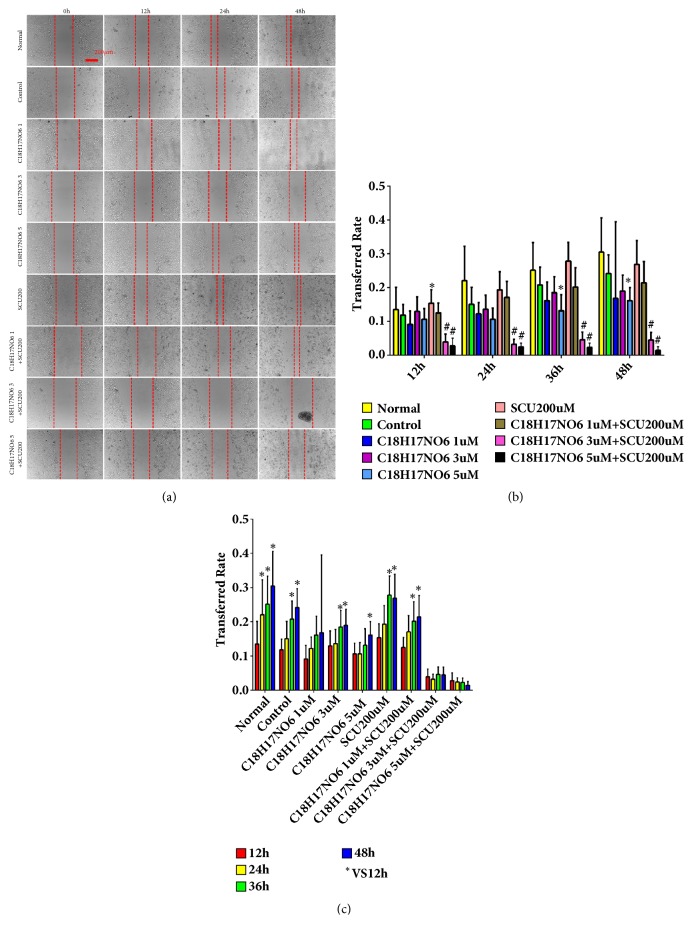
Effects of C_18_H_17_NO_6_ and its combination with Scutellarin on the transferred rate of U251 cell. (a) Photographs show the effect of C_18_H_17_NO_6_ and its combination with Scutellarin 200 *μ*M on the transfer of U251. (b) Quantification of the transferred rate of U251 in different groups at each time point. (c) Quantification of the transferred rate of U251 at different time points in each group. *∗* versus control (DMSO), # C_18_H_17_NO_6_ x versus C_18_H_17_NO_6_ x + SCU 200 *μ*M, *∗*/# P < 0.05, *∗∗*/## P < 0.01, and *∗∗∗*/### P < 0.001. The transferred rate is represented as the decimal.

The lateral transferred ability of U251 and LN229 was examined by wound healing assay. Compared with control group, C_18_H_17_NO_6_ inhibited the transferred rate of U251, but only at 36h and 48h the transferred rate of U251 in C_18_H_17_NO_6_ 5*μ*M group was statistically significant (P < 0.05) (Figures 8(a) and 8(b) and Data S8. a, b). Dramatically, Scutellarin 200 *μ*M does not reduce the transferred rate of U251. However, C_18_H_17_NO_6_ combined with Scutellarin 200 *μ*M resulted in a decrease in the transferred rate of U251 compared with C_18_H_17_NO_6_ alone. Moreover, the comparisons between C_18_H_17_NO_6_ 3 *μ*M plus SCU 200 *μ*M group and C_18_H_17_NO_6_ 3 *μ*M group and between C_18_H_17_NO_6_ 5 *μ*M plus SCU 200 *μ*M group and C_18_H_17_NO_6_ 5 *μ*M group were statistically significant (P < 0.05) (Figures 8(a) and 8(b) and Data S8. a, b). Meanwhile, with the extension of time, the transferred rate of U251 in C_18_H_17_NO_6_ 3*μ*M plus SCU 200 *μ*M group remained unchanged (P > 0.05) and decreased in C_18_H_17_NO_6_ 3*μ*M plus SCU 200*μ*M group (P > 0.05) but increased in other groups (Figures 8(a) and 8(c) and Data S8. a, b).

**Figure 9:**
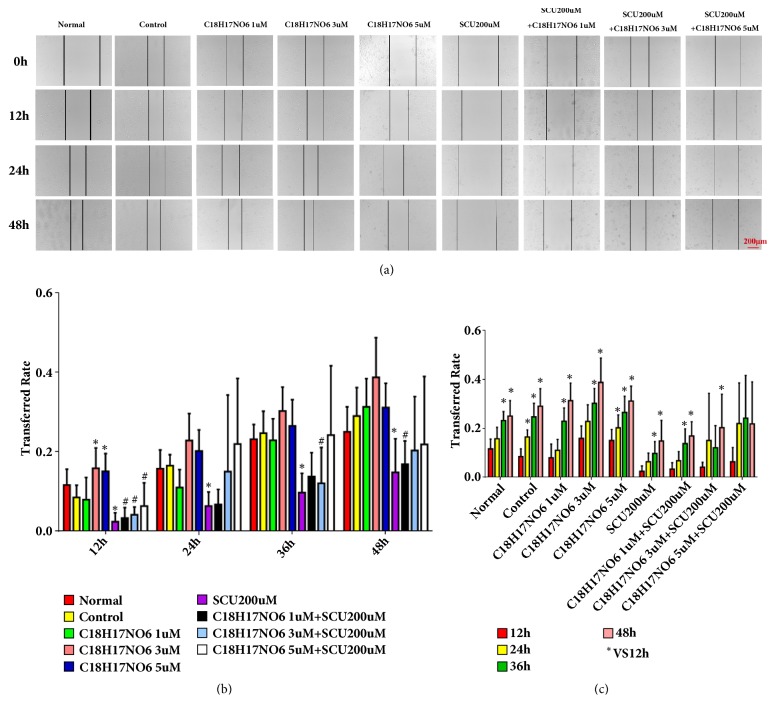
Effects of C_18_H_17_NO_6_ and its combination with Scutellarin on the transferred rate of LN229 cell. (a) Photographs show the effect of C_18_H_17_NO_6_ and its combination with Scutellarin 200 *μ*M on transfer of LN229. (b) Quantification of the transferred rate of LN229 in different groups at each time point. (c) Quantification of the transferred rate of LN229 at different time points in each group. *∗* versus control (DMSO), # C_18_H_17_NO_6_ x versus C_18_H_17_NO_6_ x + SCU 200*μ*M, *∗*/# P < 0.05, *∗∗*/## P < 0.01, and *∗∗∗*/### P < 0.001. Transferred rate is represented as the decimal.

In contrary, it was found that C_18_H_17_NO_6_ did not inhibit the transferred rate of LN229. Compared with control group, the transferred rate of LN229 in the C_18_H_17_NO_6_ groups showed an increasing trend at 12 h, 24 h, 36 h, and 48 h, but only at 12 h the differences between C_18_H_17_NO_6_ 3 *μ*M group or C_18_H_17_NO_6_ 5 *μ*M group and control group were statistically significant (P < 0.05) (Figures 9(a) and 9(b) and Data S9. a, b). However, Scutellarin 200 *μ*M significantly decreased the transferred rate of LN229 in comparison with control group, and there was a statistically significant difference at 12h, 24h, 36h, and 48h (P < 0.05) (Figures 9(a) and 9(b) and Data S9. a, b). Moreover, C_18_H_17_NO_6_ combined with Scutellarin 200 *μ*M resulted in a decrease in the transferred rate of LN229 compared to C_18_H_17_NO_6_ alone, which, but, was still higher than that of Scutellarin 200 *μ*M group (Figures 9(a) and 9(b) and Data S9. a, b). As time went by, the transferred rate of LN229 in all the groups also gradually increased (Figures 9(a) and 9(c) and Data S9. a, b).

### 2.9. The Toxic Effect of C_18_H_17_NO_6_ and Scutellarin on Astrocyte

**Figure 10:**
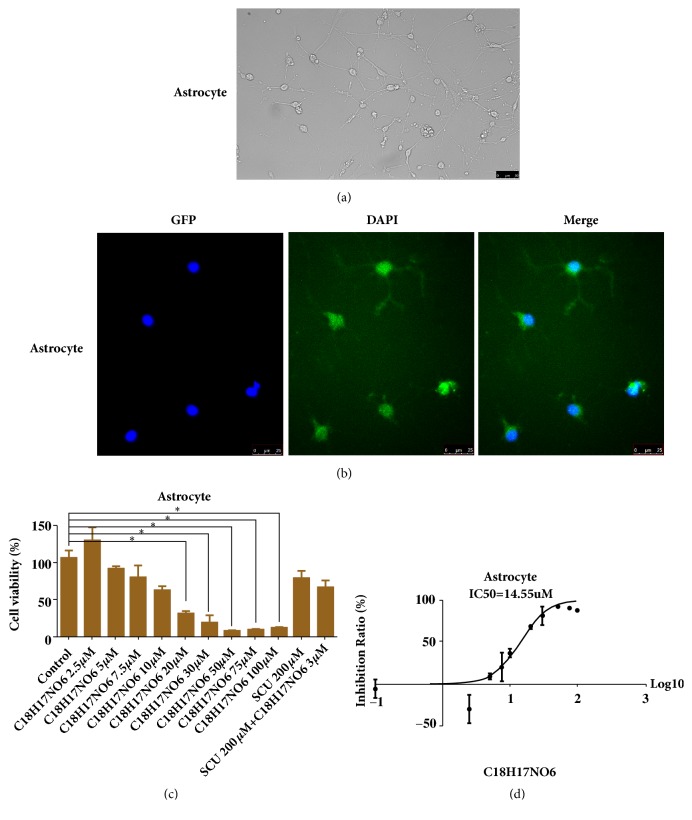
The toxic effect of C_18_H_17_NO_6_ and Scutellarin on astrocyte. (a) The bright field image shows the morphology of astrocyte. (b) The purity identification of astrocytes. (c) The effect of C_18_H_17_NO_6_ and Scutellarin on the cell viability of astrocytes. (d) The IC50 curve and IC50 of C_18_H_17_NO_6_ for astrocyte. *∗* versus control (DMSO), *∗* P < 0.05, *∗∗* P < 0.01, and *∗∗∗* P < 0.001.

In this study, the purity of cultured astrocytes was almost 90% or more (Figures 10(a) and 10(b) and Data S10. a). The cell viability of astrocyte intervened by C_18_H_17_NO_6_ and Scutellarin for 48 h was observed. We found that as the concentration of C_18_H_17_NO_6_ increased, the cell viability of astrocyte decreased, but compared with control group, the difference was statistically significant (P < 0.05) only when the concentration of C_18_H_17_NO_6_ was up to 10 *μ*M or larger ( Figure 10(c) and Data S10. b). However, there was no significant difference (P>0.05) from control group (P>0.05) when the concentration of C_18_H_17_NO_6_ was less than 10 *μ*M (Figure 10(c) and Data S10. b). Furthermore, the IC50 of C_18_H_17_NO_6_ on astrocyte was 14.55 *μ*M, with 95% confidence interval of 11.92 *μ*M to 17.77 *μ*M (Figure 10(d) and Data S10. b), which was much greater than that on U251 and LN229 cells. In our study, the maximum concentration of C_18_H_17_NO_6_ was 5 *μ*M, which had little toxic effect on astrocyte with the cell viability (91.72±3.30)% (Figure 10(c) and Data S10. b). In addition, although after intervened by Scutellarin 200 *μ*M and Scutellarin 200 *μ*M plus C_18_H_17_NO_6_ 3 *μ*M for 48 h, the cell viability of astrocyte decreased and they were (79.14±10.10)% and (67.47±8.65)%, respectively, the differences were not significantly different from control group (P > 0.05) (Figure 10(c) and Data S10. b). These results confirmed that the dose of C_18_H_17_NO_6_ and Scutellarin in this study had little toxicity to astrocyte.

### 2.10. Fas-Associated Factor 1 Was Upregulated after the Administration of C_18_H_17_NO_6_ and Its Combination with Scutellarin

**Figure 11:**
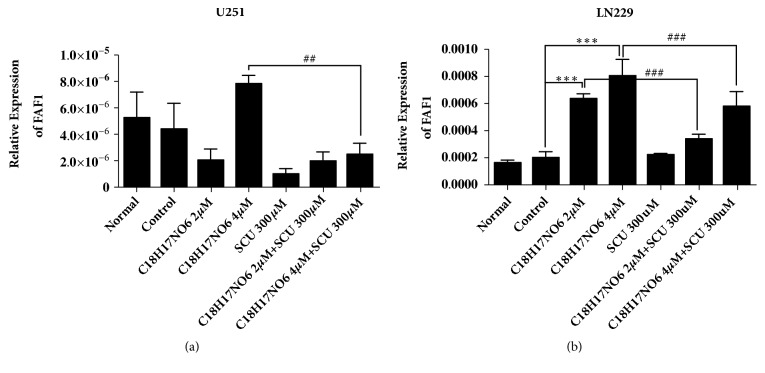
The mRNA expression of Fas-associated factor 1 (FAF1). (a) The relative mRNA expression of FAF1 in U251 cell. (b) The relative mRNA expression of FAF1 in LN229 cell. *∗* versus control (DMSO), # C_18_H_17_NO_6_ x versus C_18_H_17_NO_6_ x + SCU 300*μ*M, *∗*/# P < 0.05, *∗∗*/## P < 0.01, and *∗∗∗*/### P < 0.001.

Compared with control group, the relative mRNA expression of FAF1 in U251 cell was decreased in C_18_H_17_NO_6_ 2 *μ*M group (P>0.05), but there was an increased trend in C_18_H_17_NO_6_ 4 *μ*M group (P>0.05) (Figure 11(a) and and Data S11). Moreover, Scutellarin 300 *μ*M reduced the FAF1 mRNA expression of U251 (Figure 11(a), P>0.05, and Data S11), but had no effect on the FAF1 mRNA expression of LN229 (Figure 11(b) and Data S11). In U251 cell, the mRNA expression of FAF1 in C_18_H_17_NO_6_ and its combination with Scutellarin groups was less than that in corresponding C_18_H_17_NO_6_ alone groups (Figure 11(a) and Data S11), but was more than that Scutellarin 300 *μ*M group (Figure 11(a), P>0.05, and Data S11). In addition, with the increasing dose of C_18_H_17_NO_6_, the mRNA FAF1 expression of LN229 was gradually elevated (Figure 11(b), P < 0.001, and Data S11). However, when C_18_H_17_NO_6_ was combined with Scutellarin 300 *μ*M, the mRNA FAF1 expression of LN229 was a little lower than that in C_18_H_17_NO_6_ alone (Figure 11(b), P < 0.001, and Data S11), but still more than that in control group (Figure 11(b) and Data S11).

**Figure 12:**
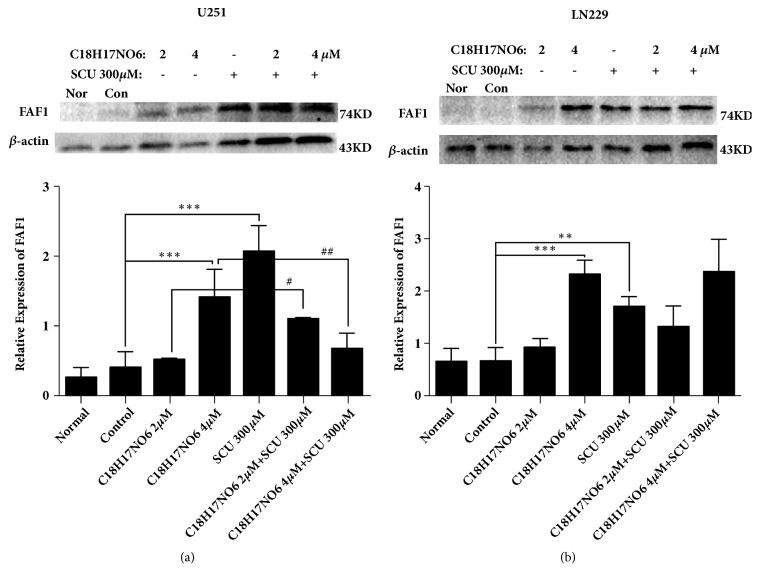
The protein expression of Fas-associated factor 1 (FAF1). (a) The relative protein expression of FAF1 in U251 cell. (b) The relative protein expression of FAF1 in LN229 cell. *∗* versus control (DMSO), # C18H17NO6 x versus C18H17NO6 x + SCU 300*μ*M, *∗*/# P < 0.05, *∗∗*/## P < 0.01, and *∗∗∗*/### P < 0.001.

For the protein level of FAF1, the increasing C_18_H_17_NO_6_ augmented FAF1 protein content in both U251 and LN229 cells (Figures 12(a) and 12(b), P < 0.001, and Data S12). In addition, the protein expression also was raised by Scutellarin 300*μ*M (Figures 12(a) and 12(b), P < 0.001, and Data S12). Moreover, the FAF1 protein expression intervened by C_18_H_17_NO_6_ and its combination with Scutellarin was more than that by C_18_H_17_NO_6_ alone in both two cells, but there was only significant statistical significance in U251 cell (Figures 12(a) and 12(b), P < 0.05, and Data S12).

## 3. Discussion

In this study, C_18_H_17_NO_6_ and its combination with Scutellarin were applied in treating glioma cells for a new therapeutic strategy. It was found that C_18_H_17_NO_6_ could efficiently inhibit cell proliferation and induce cell apoptosis in U251 and LN229 cells, and its combination with Scutellarin 200 / 300 *μ*M had a promoting effect. Besides, the upregulation of FAF1 was screened and it may refer to the underlying mechanism.

From our study, with the increasing dose of C_18_H_17_NO_6_, the cell inhibition rate was gradually increased, but the clone number and proliferation rate were gradually decreased comparing to control group. Additionally, when administrated with C_18_H_17_NO_6_, the cells in G1 phase were elevated, but the cells in G2 and S phase were lowered. All the above findings indicated that C_18_H_17_NO_6_ could effectively suppress glioma cell proliferation in vitro. Moreover, after the administration of the increasing C_18_H_17_NO_6_, the apoptosis rate of glioma cells was also found gradually increased, especially the late apoptosis rate (necrosis). However, C_18_H_17_NO_6_ could not inhibit the transfer ability, particularly in LN229 cell with an amplification of the transferred rate. C_18_H_17_NO_6_, a dibenzofuran separated from a special plant in Yunnan province (China), is a natural anticancer agent which shows strong inhibited effect on a great many cancers, but with low toxicity (patent ID: 201710388136.8). Moreover, the purity of the compound reaches 99.5% (patent ID: 201710388136.8). This drug had been explored in lung cancer, liver cancer, bladder cancer, breast cancer, nasopharyngeal carcinoma in vitro, with the IC50 1.68, 1.91, 2.11, 2.51, 3.39 *μ*M, respectively (patent ID: 201710388136.8). Furthermore, the anticancer effect is achieved by affecting cell metabolism, proliferation and cell cycle distribution (patent ID: 201710388136.8). In this study, we obtained the similar results in glioma cells to the reported in other cancers from Professor Xiao-Qiong He. Therefore, C_18_H_17_NO_6_ has potential as a therapeutic agent for the treatment of glioma.

Scutellarin is an extractant of the Chinese herbal medicine Erigeron breviscapus [23], which can expand blood vessels, improve microcirculation and anticoagulation, and have been for the treatment of cardiovascular and cerebrovascular diseases [24–27]. However, it has also been reported that it has antitumor effect in various tumors. First, Scutellarin reduced the viability and induced cell death in human colorectal cancer cells by regulating p53 and Bcl 2/Bax expression [28] and a network of proteins involved in metabolism, regulation of the cell cycle, and transcription-factor activity [29]. Then, Scutellarin also inhibit the proliferation and inhibit the lung and intrahepatic metastasis and migration and invasion of hepatocellular carcinoma in vitro by down-regulating the STAT3/Girdin/Akt signaling [30, 31]. Moreover, Scutellarin showed effect to suppress the proliferation and promote the apoptosis on tongue squamous carcinoma through the inhibition of matrix metalloproteinase-2 and -9 (MMP-2, MMP-9) and *α*v*β*6 integrin [32]. Additionally, Scutellarin could dysregulate the apoptosis and cell cycle of leukemia cell and Burkitt lymphoma Namalwa cell [33, 34]. In this study, Scutellarin 200 *μ*M have little effect on proliferation, with the inhibition rate 10-20% and proliferation rate similar to that in control group. In addition, compared to control group, Scutellarin 300 *μ*M increased the U251 cells in G2 and S phases, but decreased the LN229 cells in G2 and S phases. Besides, Scutellarin 200 *μ*M also could not efficaciously induced the apoptosis of U251 and LN229 cells. When the dose of Scutellarin was add to 300 *μ*M, the apoptosis of LN229 was augmented about 75% in comparison to control group, but similar to control group in U251 cell. In Scutellarin 200 *μ*M group, the transfer ability of LN229 was inhibited, but not in U251. Furthermore, if C_18_H_17_NO_6_ was combined with Scutellarin 200 / 300 *μ*M, the proliferation and transferred ability was suppressed and the apoptosis was elevated in LN229 cell in comparison with C_18_H_17_NO_6_ alone. Dramatically, the combined effect on U251 was the exact opposite. These results suggested that the effect of Scutellarin 200 / 300 *μ*M on U251 and LN229 cells was ambiguous. Perhaps, the essential reason is that the status of the gene alterations of U251 and LN229 cells is different, which results in different response to Scutellarin. As you know, PTEN frameshift mutation was identified in U251 cell, but in LN229 [35, 36]. In addition, the p53 mutation of U251 located at 273th codon with transforming CGT(Arg) into CAT(His), but at 98th codon with transforming CCT(Pro) into CTT(Lys) [35]. Although p16 and p14ARF deletion existed in both U251 and LN229 cells, the mutation sites are different [35]. In this paper, the concentration of Scutellarin for U251 cells might be too low to show its pharmacodynamic effect.

The FAF1 was screened in The Cancer Genome Atlas (TCGA) (https://cancergenome.nih.gov). Not only Fas-associated factor (FAF)-1 was a member of the Fas death-inducing signaling complex, but it was involved in various biological processes and played an essential role in cancer, asbestos-induced mesotheliomas, and Parkinson's disease [37, 38]. In especial, FAF1, reported as a tumor suppressor [39], was found to decrease in many cancers, such as gastric carcinomas [40] and human breast carcinoma [41]. Elmetwali T et al. found that FAF1 suppressed CD40-induced NF*κ*B activity via a negative feedback loop [42]. In addition, miR-24 was found to target Fas-associated factor 1 (FAF1) by binding to its amino acid coding sequence (CDS) region to regulated apoptosis in hormone-insensitive prostate cancer or other types of cancers [43]. Moreover, SNG, a benzophenanthridine alkaloid isolated from the Papaveraceae plants, increased Fas-associated factor 1 expression, which inhibited cell proliferation, invasion, and migration and induced cell cycle arrest and apoptosis of non-small-cell lung cancer (NSCLC) in vitro and in vivo [44]. Conversely, the completely opposite result was acquired by knockdown of FAF1 [44]. In this study, the FAF1 expression of U251 and LN229 was upregulated by C_18_H_17_NO_6_ and its combination with Scutellarin. This result linking with the observed effects on glioma cells by C_18_H_17_NO_6_ and its combination with Scutellarin suggested that the underlying mechanism of these drugs might be upregulation of FAF1, which might be a valid therapeutic target and provide a novel mechanism by which to treat glioma.

In addition, we found that the low toxicity of C_18_H_17_NO_6_ and Scutellarin in the rat astrocyte. Here, rat astrocyte was used as research object because of their ease of acquisition and their ability to investigate the toxicity of both drugs. At first, astrocyte is the most abundant and widely distributed glial cells in nerve tissue [45]. Since the peak of astrocyte mitosis is in the late stage of animal embryos and after birth, it is usually appropriate to select the astrocytes of newborn animals [46, 47]. If the astrocytes from neonatal rat cerebral cortex are inoculated at a lower density, high purity astrocytes can be cultured. Secondly, glioma originates from the amplification of mutant astrocytes [48], but human primary astrocytes are difficult to obtain. Therefore, the toxicity analysis of the two drugs took rat astrocytes as the object. In addition, rat astrocytes express a large amount of GFAP protein, while other cells of the nervous system do not express this protein [46, 49, 50]. Thus, whether GFAP protein was expressed or not could be used for the purity identification of astrocytes.

## 4. Materials and Methods

### 4.1. Materials

U251 and LN229 cell lines were provided by Professor Jia Geng, National Key Laboratory of Sichuan University. The complete medium was compounded by 89% of DMEM high-glucose medium (Hyclone), 10% fetal bovine serum (Gibco), and 1% double antibiotics (Hyclone); 0.25% trypsin (Hyclone), C_18_H_17_NO_6_ (provided by Professor Xiao-Qiong He, Kunming Medical University), Scutellarin (provided by the School of Pharmacy, Kunming Medical University), 0.2 *μ*m millipore filter (Millipore), Dimethyl Sulphoxide (DMSO, Sigma), Cell Counting Kit-8 (DOJINDO, Japan), the CO_2_ incubator (Thermo), Microplate Spectrophotometer (Thermo), Centrifugal Machine (TD-4Z, Shuke Instrument Co., Ltd, Sichuan, China), Pipette tips (Axygen), Centrifuge tubes (Corning), and Culture dishes (Corning); Ophthalmic scissors, Ophthalmic forceps, and Micro forceps (Hong Bang Medical Equipment Co., Ltd., Jiangsu, China); 25 cm_2_ culture flask and culture plates (Corning), Ultra clean cabinet (Thermo), Ultra-pure water equipment (Type: GN-RO-40, Shuangfeng Pure Water Equipment Factory, Beijing, China), Anatomical microscope (Nikon), Inverted fluorescence microscope (Nikon), Cell Insight CX5 (Thermo), the incubator with constant temperature (Type: KB115, BINDER, Germany), micropipettors (Ependorf), 75% medical alcohol, 0.01M Phosphate buffer solution (PBS, Hyclone), 1% crystal violet (beyotime), Cell-Light™ EdU Apollo®567 In Vitro Imaging Kit (RiboBio, Guangzhou, China), 2 mg/mL glycine (Sigma), TritonX-100 (Sigma), methanol (Guanghua Sci-Tech Co., Ltd., Guangdong, China), In Situ Cell Death Detection Kit, TMR red (Roche), flow cytometry (ACEA, Novo Express International, Inc., China), Propidium Iodide/Ribonuclease staining solution (BD Bioscience), Annexin-V-FITC Apoptosis Detection Kit (Beyotime), TRIzol™ Reagent (Invitrogen), Revert Aid TM First Strand cDNA Synthesis Kit (Thermo Scientific), C1000 Touch™ Thermal Cycler (BIO-RAD), Goat serum (Invitrogen), GFAP primary antibody (Rabbit, Proteintech, 1:100), secondary antibody Alexa488 (anti-rabbit, Invitrogen, 1:200), DAPI (Beyotime), BCA Protein Assay Kit (Beyotime), 5X SDS-PAGE protein sample buffer (Biosharp), anti-FAF1 primary antibody (Rabbit, 1:500, Bioss) and anti-*β*-actin primary antibody (Mouse, 1:2000, Abbkine), peroxidase-conjugated goat anti-mouse/rabbit IgG secondary antibody (1:5000, Abbkine), and Molecular Imager ChemiDoc™ XSR+ Imaging System (BIO-RAD).

### 4.2. Cell Culture

#### 4.2.1. Cell Lines

The glioma cell lines U251 and LN229 were cultured in complete medium at 37°C in the incubator containing 5% CO_2_. When the degree of fusion of the cells reached 80 to 90%, they were digested with 0.25% trypsin for 3-5 minutes. After the cells were observed to be rounding under the microscope, the equal amount of complete medium to trypsin was used to stop the digestion. Then the cells were suspended by gently pipetting, and the cell suspension was collected in centrifuge tubes and centrifuged at 1000 rpm for 5 minutes. The supernatant was discarded. Finally, the complete medium was added to resuspend the cells and prepare the cell suspension for inoculation or subsequent experiments.

#### 4.2.2. Primary Astrocyte

Neonatal 1-3 day SD rats were soaked in 75% alcohol for 1 minute, then decapitated and exposed the entire brain. The brain was completely taken out. After washing in 0.01M PBS, the meninge was carefully dissected under the anatomical microscope, and the cortical tissue was carefully harvested using a microforceps. Then the cortical tissue was cut into 1 mm3 tissue pieces and digested by 0.25% trypsin (1ml/mouse) at 37°C for 15 min. The equal amount of complete medium was used to stop the digestion. The cells were gently pipetted several times and collected into centrifuge tubes through a millipore filter to centrifuge at 1000 rpm for 10 minutes. After that, the supernatant was discarded and fresh complete medium was added to prepare 2~5×10^5^/ml single cell suspension. This single cell suspension was inoculated into 25cm_2_ culture flasks (5ml/flask) and incubated at 37°C in a 5% CO_2_ incubator. Then the medium was changed once every 3 days. On the seventh to ninth day, it was observed that the degree of cell fusion reached 80% and then the cells were shaken overnight on a shaker with 200 rpm to purify the astrocytes. The astrocytes were then digested with 0.25% trypsin to make a single cell suspension and inoculated. After purification, P1 generation of astrocytes was passaged and immunofluorescence staining of GFAP was performed to identify astrocyte purity. When the purity was 90% or more, the cells were used for subsequent experiments.

### 4.3. Cell Viability Analysis

When the glioma cell lines U251 and LN229 were grown to the logarithmic phase, the collected cells were seeded in 96-well plates at 3000-5000 cells per well. After cultured overnight at 37°C in a 5% CO_2_ cell incubator, the cells were administrated with different concentration of C_18_H_17_NO_6_ and its combined with Scutellarin for 24, 48, 72h. Then add 10 ul of CCK8 reagent to each well and incubate for 4 h. The absorbance (OD value) was used by a microplate spectrophotometer at 450 nm. In the study, the inhibition rate = (Ac-As)/(Ac-Ab)×100% and cell viability = (As-Ab)/(Ac-Ab)×100%. The meaning of the parameters is as follows. As is the absorbance of drug intervention groups, Ac is the absorbance of control group (that is the solvent (DMSO) group), and Ab is the absorbance of blank control group.

In addition, for the cell viability assay of primary astrocytes, 10,000 cells/well were seeded in the 96-well plates, and the remaining steps were the same as above.

### 4.4. Plate Clone Formation Assay

On the first day, 1000 wells were inoculated into each well of the 6-well culture plates, and 3 replicate wells were set in each group. After the cells were adhered overnight in the 37°C incubator, the drugs were added and the cells were further cultured in the 37°C incubator until the 14th day. Meanwhile, the medium was changed several times and the cell state was also observed. Before the termination of the experiment, cell clones were photographed under a fluorescence microscope, and the number of cells per clone was counted to be greater than 50. Then, the cells were washed 3 times with 0.01M PBS, 5 min/time. At once, the cells were fixed with 4% paraformaldehyde for 15 minutes, and the cells were washed 3 times with 0.01M PBS, 5 min/time. After that, 0.5ml of 0.5% crystal violet was added to each well to dye for 10 min. Then the cells were washed 3 times with 0.01M PBS, 5 min/time. At last, the pictures were acquired with a camera, and the number of clones was counted.

### 4.5. EdU Incorporation Assay

The cells in logarithmic phase were seeded in the 96-well plates with 5000 cells per well and cultured to normal growth stage. At this time, the cells were subjected to drug treatment for 48 hours. Then the EdU was diluted to 50 *μ*M EdU solution with the complete medium and 100 *μ*l was added per well to incubate for 2 hours. After washing the cells twice with 0.01M PBS, 5 min/time, 50 *μ*l of 4% paraformaldehyde was added per well to fix the cells for 30 minutes at room temperature, then the fixation fluid was discarded, and 50 *μ*l of 2 mg/ml glycine was added to each well to incubate for 5 minutes on the shaker so as to neutralize excess aldehyde and 0.01M PBS was used for washing for 5 minutes. Then each well was added 100*μ*L of 0.5% TritonX-100 in 0.01M PBS to incubate for 10 minutes on a shaker and washed with 0.01M PBS for 5 minutes. Next, 100 *μ*L of 1×Apollo® staining solution was added to each well and incubated for 30 minutes on the shaker at room temperature (pay attention to the light). After discarding the staining solution, washing was performed with the following in turn. (1) 0.5% TritonX-100 in 0.01M PBS for 3 times on the shaker, 10min/time. (2) methanol for 2 times, 5min/time. (3) 0.01M PBS for 5min. Finally, 1× Hoechst33342 reaction solution was prepared and 100 *μ*l was added to each well to incubate for 30 minutes in the dark at room temperature. After discarding and washing 3 times with 0.01M PBS, 5 minutes/time, the images were acquired with Thermo Cell Insight CX5. At the same time, the EdU positive cell number and the Hoechest positive cell number of each well were count and analyzed by Thermo Cell Insight CX5. The proliferation rate = EdU positive cell number (red)/Hoechest positive cell number (blue)×100% was calculated.

### 4.6. Cell Apoptosis Analysis by TUNEL Assay

The cells were seeded in the 96-well plates with 5000/well and adhered overnight. Drugs were administrated for 48 hours, and the supernatant was removed and washed 3 times with 0.01M PBS, 5 min/time. Then the cells were fixed with 4% paraformaldehyde for 15 min at 15-25°C and wash 3 times with 0.01M PBS, 5 min/time. After that, the cells were permeabilized by sodium citrate solution containing 0.1% Triton X-100 for 2 minutes on the ice (2-8°C), and washed twice with 0.01M PBS, 5 min/time. Then we mixed 50 *μ*l TdT with 450 *μ*l fluorescein-labelled dUTP solution to prepare the TUNEL reaction mixture, and the prepared TUNEL reaction mixture was performed then added to the plate well with 50 *μ*l/well to incubate for 1 hour at 37°C in the dark. After washing, 50*μ*l of 5*μ*g/ml DAPI reaction solution was added to each well and incubate for 5 min at room temperature in the dark. By using Thermo Cell Insight CX5, the images were obtained, and the TUNEL- and DAPI-labelled cells were counted, respectively. The apoptosis rate = TUNEL-positive cells (red)/DAPI-positive cells (blue)×100%.

### 4.7. Detecting Cell Cycle and Apoptosis by Flow Cytometer

Experimental groups: control group (DMSO), C_18_H_17_NO_6_ 2 *μ*M group, C_18_H_17_NO_6_ 4 *μ*M group, SCU 300 *μ*M group, C_18_H_17_NO_6_ 2 *μ*M + SCU 300 *μ*M group, C_18_H_17_NO_6_ 4 *μ*M + SCU 300 *μ*M group.

For the detection of cell cycle, the cells were gently collected after 48 h of drug intervention. This was performed a centrifugation and the supernatant was discarded. In 1×10^6^ cells, 1 ml of precooling 70% ethanol at 4°C was slowly added to fix the cells. In order to reduce cell aggregation, the centrifuge tubes were shook while adding 70% ethanol. The cells were fixed overnight at 4°C, and then centrifuged at 1000 rpm for 5 min. The fixative was discarded, and 2 ml of 0.01 M PBS was added to wash the cells twice. The cells were resuspended with 500 ul of PI/ribonuclease staining solution and stained for 15 min at room temperature in the dark. Finally, according to the standard protocol, more than 10,000 cells were detected by flow cytometry.

For cell apoptosis detection, the experimental design included the compensation adjustment groups and the experimental groups. The compensation groups were blank group (nonstained), Annexin-V single-staining group and PI single-staining group. All the compensation groups were induced apoptosis by heat shock method, in which, the cells were incubated in a water bath at 43°C for 1 h and allowed to stand for 24 h [51]. In addition to the above groups, the experimental group also included positive control (heat shock induced apoptosis group). The specific procedure was as follows. the cells were digested after 48 hours of drug treatment with 0.25% trypsin and collected to make a single cell suspension. Then wash twice with 0.01 M PBS. Subsequently, 100 ul of the binding buffer and 10 ul of 20 ug/ml FITC-labelled Annexin-V were added to incubate for 30 minutes at room temperature in the dark. Then 10 ul of 50 *μ*g/ml PI was added to react for 5 minutes in the same condition. Finally, 400 *μ*l of binding buffer was added and then detected by a flow cytometer according to standard protocol, in which flow cytometer would collect 20000 to 30000 cells per test. During the process, the live cells were not stained with Annexin-V and PI, the early apoptotic cells were only stained with Annexin-V, and the late apoptotic/necrotic cells were stained with Annexin-V and PI, and mechanically damaged cells were only stained with PI.

### 4.8. Wound Healing Assay

Wound healing assay was used to evaluate the transfer ability of cells. About 1×10^6^ cells were added to each well of the six-well plates, and cells were spread over the bottom of the well overnight. Using a 10 ul pipette, draw a straight “#” across the plate well, and then wash it 3 times with DMEM high-glucose medium to remove the detached cells. Then fresh complete medium containing the drug was added and placed in a 37°C incubator containing 5% CO_2_. At 0h, 12h, 24h, 36h, and 48h, the images of 10 fields in each group were taken at the same location with an inverted microscope. Moreover, IPP 6.0 software was used to measure the area of each field at 0h, 12h, 24h, 36h, and 48h and the average scratch distance of each field is equal to the area divided by the length (here the scratch length of each field are the same). The transfer distance of the cells was then equal to the difference between the average distance at 12h, 24h, 36h, and 48h and the average distance at 0h. The transferred rate = (the average distance at 0h - the average distance at x h)/(the average distance at 0h)×100%.

### 4.9. Quantitative Reverse Transcription Polymerase Chain Reaction (qRT-PCR)

According to the series of Fas-associated factor 1 published in GenBank, the primers were designed by Primer 5.0 software and synthesized by Takara Biotechnology Co., Ltd. The sequence is shown in Table 1, containing forward primer (restriction enzyme site-Hind included) and reverse primer. To extract total RNA, 1 ml TRIzol reagent was added per 1×10^6^ cells, and total RNA was extracted according to the reference standard. After the obtained RNA was dissolved, 2.5 *μ*l of RNA was taken out for quality inspection. The extracted RNA was then reversely transcribed into cDNA with random primers and reverse transcriptase according to the instructions of Revert AidTM First Strand cDNA Synthesis Kit. In addition, using the cDNA as a template and *β*-actin as an internal reference, the cDNA was amplified in a Revert AidTM Frist Strand cDNA kit (Fermentas). The cycling conditions were 95°C for 3 minutes, 95°C for 15 seconds, 72°C for 30 seconds, 45 cycles and 60°C for 30 seconds. Calculate and determine the amount of template based on the standard curve. Threshold cycles (Ct values) were recorded for each sample and the data normalized to *β*-actin value were analyzed using the 2^-ΔΔCt^ method.

### 4.10. Western Blot Analysis

The total protein of U251 and LN229 cells intervened for 48h by C_18_H_17_NO_6_ and its combination with Scutellarin were extracted by 98% RIPA lysis buffer (Beyotime) and 2% cocktail pill (Roche). The lysate were centrifuged to obtain supernatant. Then the protein concentration was determined by the BCA Protein Assay Kit (Beyotime). After the supernatant was mixed with 1X SDS-PAGE protein sample buffer (Biosharp), proteins were separated by sodium dodecyl sulfate-polyacrylamide gel (SDS-PAGE) electrophoresis (10%) and transferred to the polyvinylidene fluoride (PVDF) membranes. The PVDF membranes were blocked with 5% nonfat milk into 1×TBS (Tris-buffered saline: 50 mM Tris, 150 mM NaCl, pH 7.6) at room temperature for 2h. After that, the membranes were probed overnight with 5% BSA buffer containing anti-FAF1 primary antibody (Rabbit, 1:500, Bioss) and anti-*β*-actin primary antibody (Mouse, 1:2000, Abbkine) at 4°C and then incubated with horseradish peroxidase-conjugated goat anti-mouse/rabbit IgG secondary antibody (1:5000, Abbkine). ECL chemiluminescent substrates (Beyotime) were as substrates of the horseradish peroxidase and the detection was performed using Molecular Imager ChemiDoc™ XSR+ Imaging System (BIO-RAD). Band intensity was quantified by ImageJ software (NIH). The ratio of the average intensity of FAF1 and that of *β*-actin was used for FAF1 semiquantitative analysis.

### 4.11. Immunofluorescence Staining

The primary astrocytes were washed with 0.01M PBS three times after 48h drug intervention. Then the cells were fixed with 4% paraformaldehyde for 15 min at room temperature, and washed three times with 0.01M PBS. In order to block nonspecific binding or reduce nonspecific background, 5% goat serum containing 0.5% Triton X-100 was added to incubate for 30 minutes at room temperature. Next, the anti-GFAP primary antibody (rabbit, Proteintech, 1:100) was added to the wells and the plates were placed in a wet box and incubated overnight at 4°C. After washing 3 times with 0.01 M PBS, the secondary antibody labelled by Alexa488 fluorescein (anti-rabbit, Invitrogen, 1:200) was added and incubated at 37°C in the dark for 1 hour. Next, the cells were washed 3 times with 0.01M PBS in the dark and the cell nuclei were stained with 2 *μ*g/ml DAPI for 10 min at room temperature later. Finally, the images were observed and obtained by an immunofluorescence microscope.

### 4.12. Statistical Analysis

Data was represented as mean ± SD. The data from three independent groups and above was analyzed by one-way ANOVA. General linear model-repeated measures were used for the repeated measurement data analysis. All data were analyzed using SPSS 16.0 software (IBM Corporation, Armonk, NY, USA). *∗*/# meant P<0.05, *∗∗*/## meant P<0.01, and *∗∗∗*/### meant P<0.001. As long as P<0.05, the difference was considered to be statistically significant.

## 5. Patents

C_18_H_17_NO_6_, a dibenzofuran separated from a special plant in Yunnan province (China), is a new natural anticancer agent with low toxicity (patent ID: 201710388136.8).

## Figures and Tables

**Table 1:** The primers sequence of Fas-associated factor 1.

Primers	**Sequence**
Forward	GTCTTTATGTCCTTACACCAG
Reverse	GGTGATGATCAGCATGAAGT

## Data Availability

All data generated or analyzed during this study are included in this published article and its supplementary information files.

## Supplementary Materials

Supplementary Materials Supplementary Data 1: (a) the inhibition rate of C_18_H_17_NO_6_ on glioma cells by CCK8 test; (b) the inhibition rate of Scutellarin on glioma cells by CCK8 test. Supplementary Data 2: (a) the cell viability of glioma cells intervened by C_18_H_17_NO_6_ and its combination with Scutellarin for 24h with cck8 test, (b) the cell viability of glioma cells intervened by C_18_H_17_NO_6_ and its combination with Scutellarin for 48h with cck8 test, and (c) the cell viability of glioma cells intervened by C_18_H_17_NO_6_ and its combination with Scutellarin for 72h with cck8 test. Supplementary Data 3: the clone count of LN229 cell intervened by C_18_H_17_NO_6_ and its combination with Scutellarin for 48h. Supplementary Data 4: (a) the effect of C_18_H_17_NO_6_ and its combination with Scutellarin on the proliferation of U251-the figures of EdU incorporation assay, (b) the effect of C_18_H_17_NO_6_ and its combination with Scutellarin on the proliferation of LN229-the figures of EdU incorporation assay, and (c) the effect of C_18_H_17_NO_6_ and its combination with Scutellarin on the proliferation rate of glioma cells by EdU incorporation assay. Supplementary Data 5: the effect of C_18_H_17_NO_6_ and its combination with Scutellarin on the cell cycle of glioma cells by flow cytometry analysis. Supplementary Data 6: (a) the effect of C_18_H_17_NO_6_ and its combination with Scutellarin on the apoptosis of U251-the figures of TUNEL assay, (b) the effect of C_18_H_17_NO_6_ and its combination with Scutellarin on the apoptosis of LN229-the figures of TUNEL assay, and (c) the effect of C_18_H_17_NO_6_ and its combination with Scutellarin on the apoptosis rate of glioma cells by TUNEL assay. Supplementary Data 7: the effect of C_18_H_17_NO_6_ and its combination with Scutellarin on the apoptosis rate of glioma cells by flow cytometry analysis Supplementary Data 8: (a) the effect of C_18_H_17_NO_6_ and its combination with Scutellarin on the lateral transferred ability of U251-the figures of wound healing assay; (b) the effect of C_18_H_17_NO_6_ and its combination with Scutellarin on the transferred rate of U251 cells by wound healing assay. Supplementary Data 9: (a) the effect of C_18_H_17_NO_6_ and its combination with Scutellarin on the lateral transferred ability of LN229-the figures of wound healing assay; (b) the effect of C_18_H_17_NO_6_ and its combination with Scutellarin on the transferred rate of LN229 cells by wound healing assay. Supplementary Data 10: (a) the immunofluorescence staining and bright field pictures of normal astrocytes; (b) the toxic effect of C_18_H_17_NO_6_ and its combination with Scutellarin on astrocytes by CCK8 analysis. Supplementary Data 11: the mRNA expression of FAF1 in glioma cells after intervened by C_18_H_17_NO_6_ and its combination with Scutellarin for 48h. Supplementary Data 12: the protein level of FAF1 in glioma cells after being intervened by C_18_H_17_NO_6_ and its combination with Scutellarin for 48h.
