# Correction to “C_18_H_17_NO_6_ and Its Combination with Scutellarin Suppress the Proliferation and Induce the Apoptosis of Human Glioma Cells via Upregulation of Fas‐Associated Factor 1 Expression”

**DOI:** 10.1155/bmri/9810459

**Published:** 2026-07-07

X.‐Y. He, L.‐L. Xiong, Q.‐J. Xia, et al., “C_18_H_17_NO_6_ and Its Combination with Scutellarin Suppress the Proliferation and Induce the Apoptosis of Human Glioma Cells via Upregulation of Fas‐Associated Factor 1 Expression,” *BioMed Research International,* 2019, no. 1 (2019): 6821219, https://doi.org/10.1155/2019/6821219.

In the article, errors were identified in the western blots shown in Figure 12 [1]. The authors then contacted the publisher to correct the figure. After assessment of the author′s request and original files, the editorial board confirmed that the overall results and conclusions are not affected and that the figure should be corrected.

The correct Figure 12 is shown below, which contains corrected *β*‐actin blots and quantification measurements.

**Figure 12:**
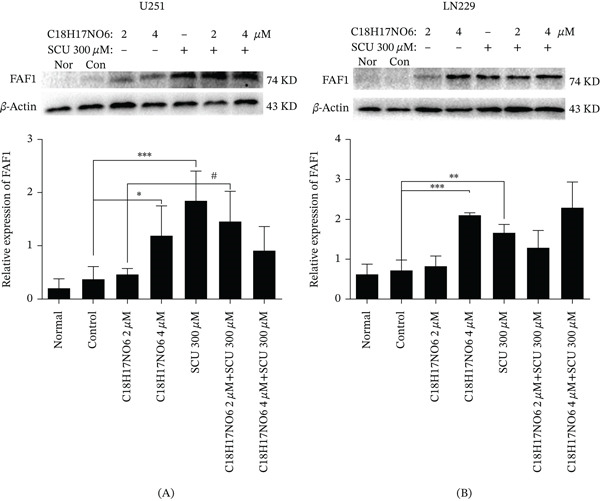
The protein expression of Fas‐associated factor 1 (FAF1). (A) The relative protein expression of FAF1 in U251 cell. (B) The relative protein expression of FAF1 in LN229 cell. ∗ versus control (DMSO), #C_18_H_17_NO_6_ x versus C_18_H_17_NO_6_ x + SCU 30 μM, ∗/#*p* < 0.05, ∗∗/##*p* < 0.01, and ∗∗∗/###*p* < 0.001.

We apologize for this error.

## References

[1] Fosteriana T. and Bik E. , C_18_H_17_NO_6_ and Its Combination With Scutellarin Suppress the Proliferation and Induce the Apoptosis of Human Glioma Cells via Upregulation of Fas-Associated Factor 1 Expression, PubPeer. (2021) https://pubpeer.com/publications/823EAB432C62FA6F7FB91B983B8F5F. 10.1155/2019/6821219 PMC6402243 30915356

