# Supplementary material for: C18H17NO6 and Its Combination with Scutellarin Suppress the Proliferation and Induce the Apoptosis of Human Glioma Cells via Upregulation of Fas-Associated Factor 1 Expression
Source: Biomed Res Int. 2019 Feb 20;2019:6821219. doi: 10.1155/2019/6821219 (PMC6402243; doi:10.1155/2019/6821219)

**1. Normal**

0h:


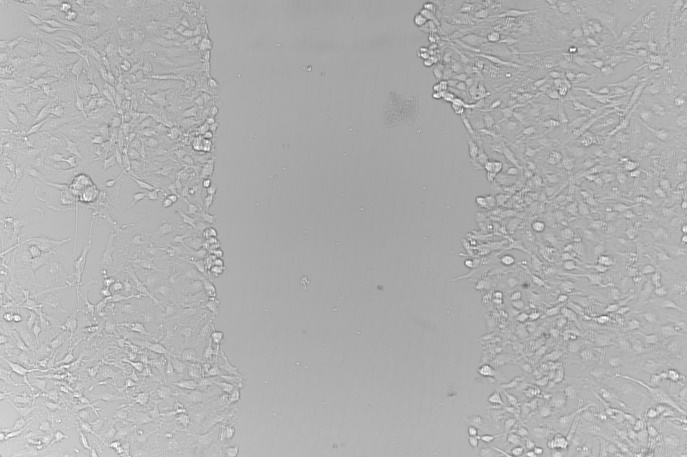


12h:


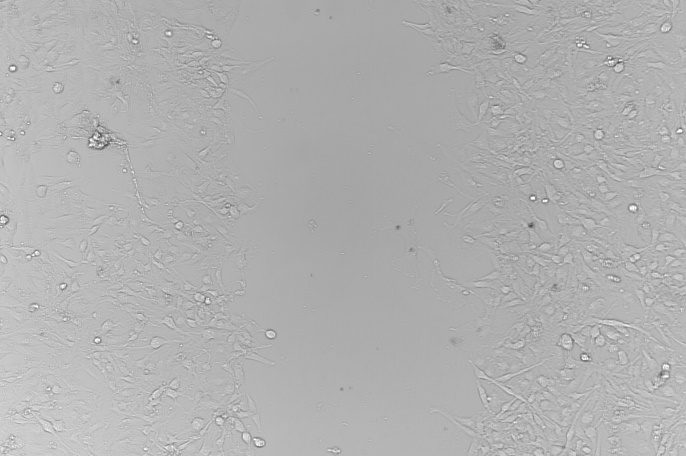


24h:


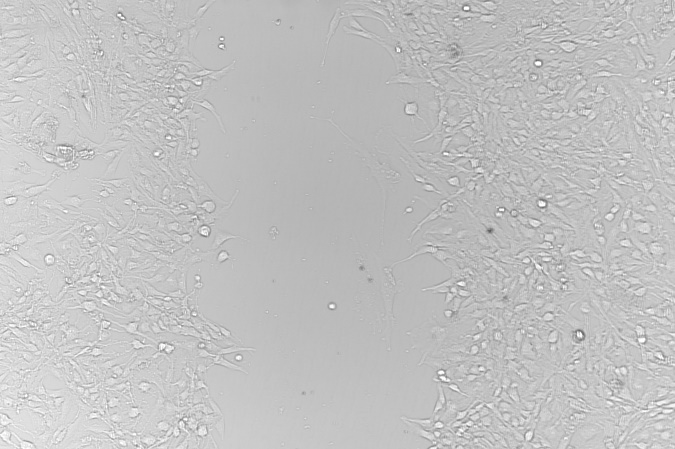


36h:


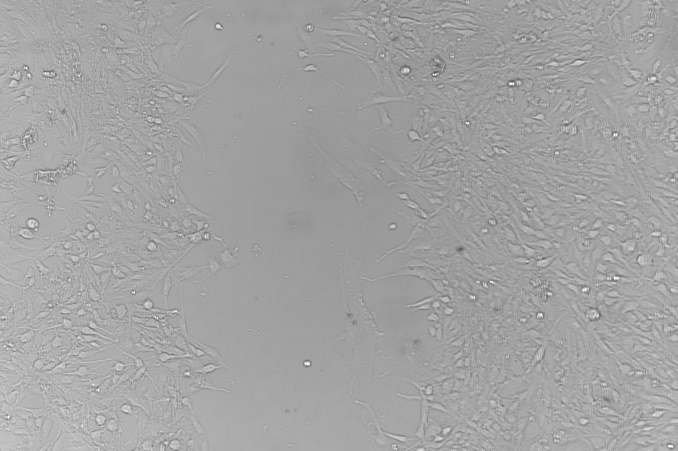


48h:


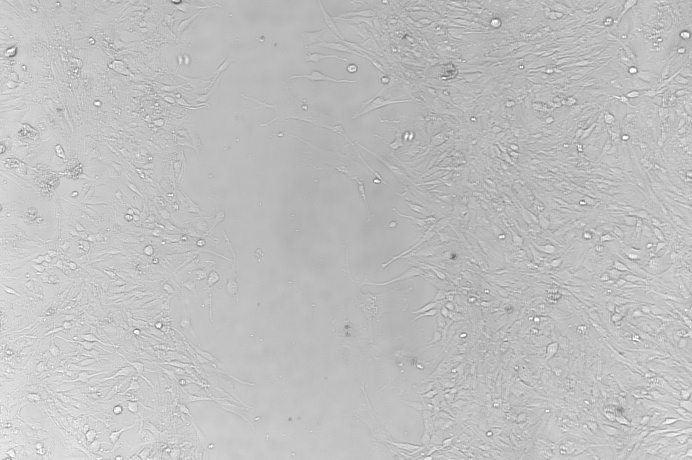


**2. Control**

0h:


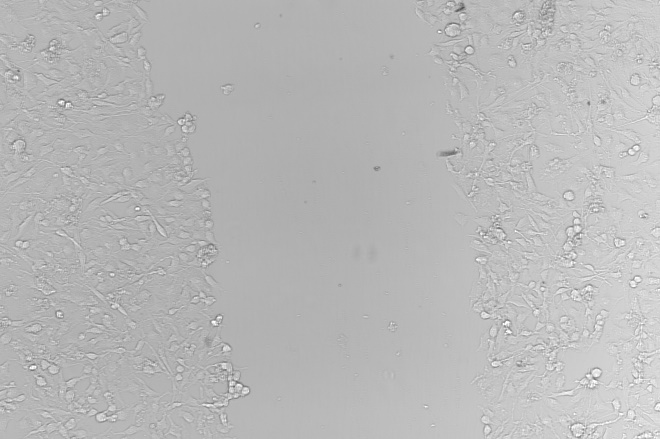


12h:


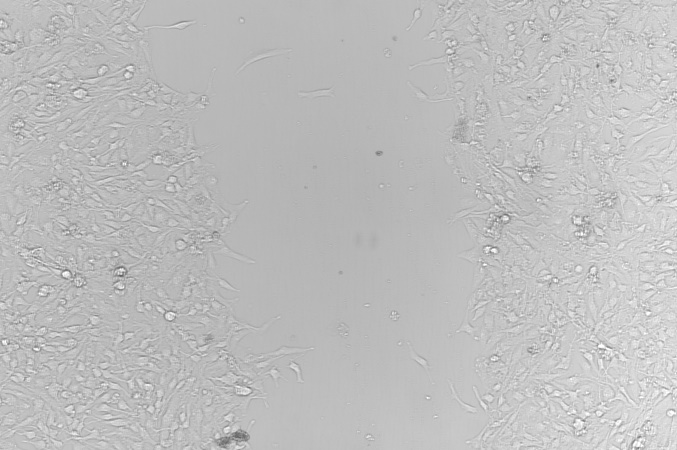


24h:

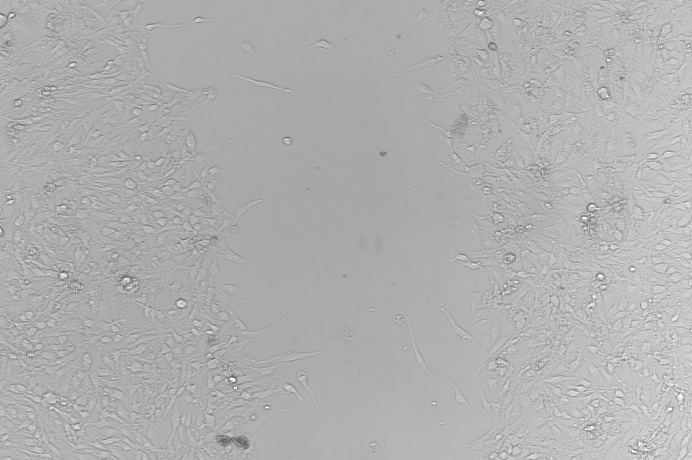


36h:


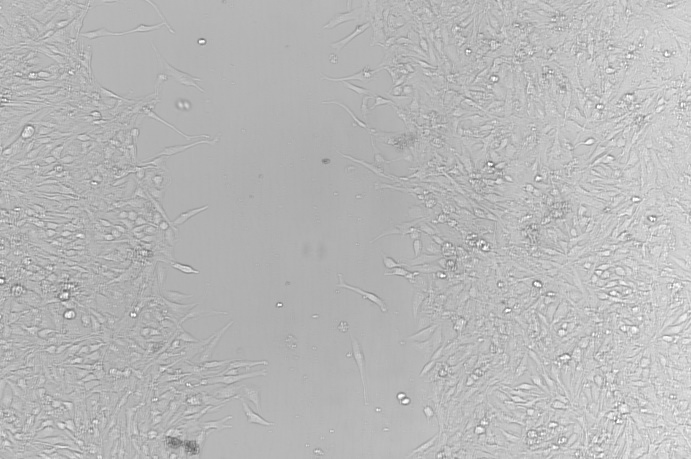


48h:


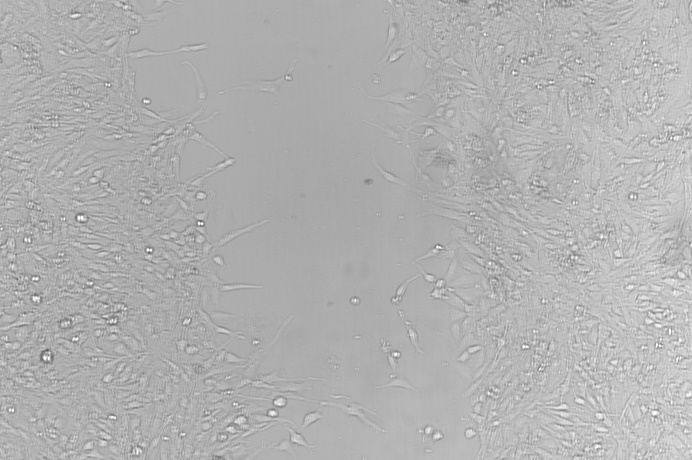


**3. C_18_H_17_NO_6_ 1μM**

0h:


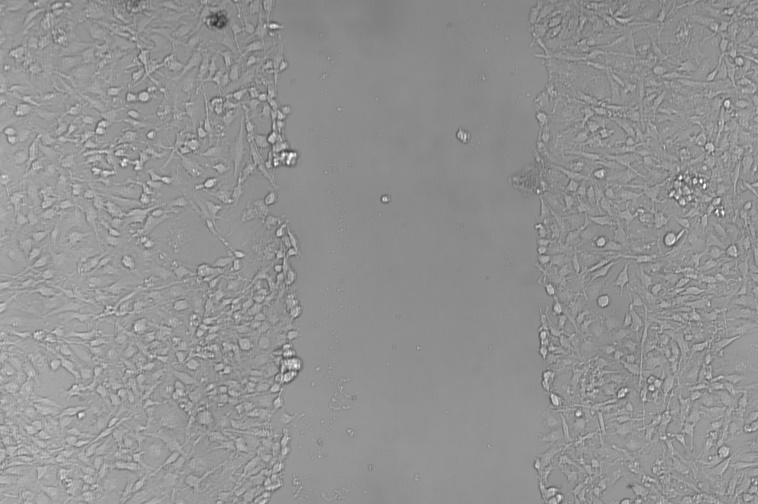


12h:


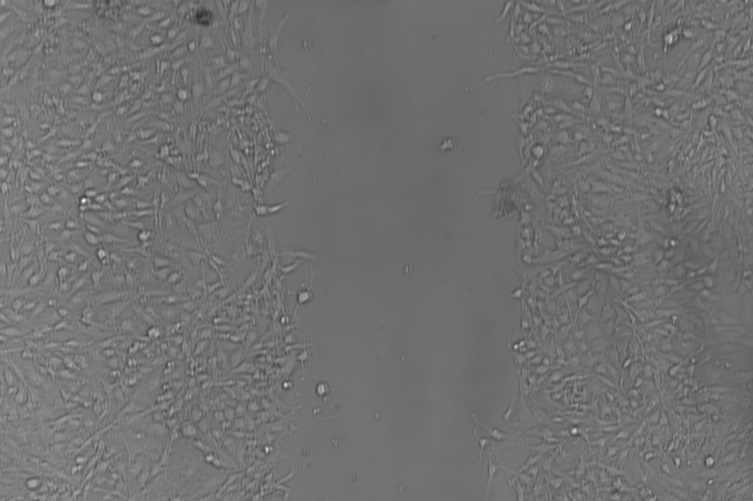


24h:


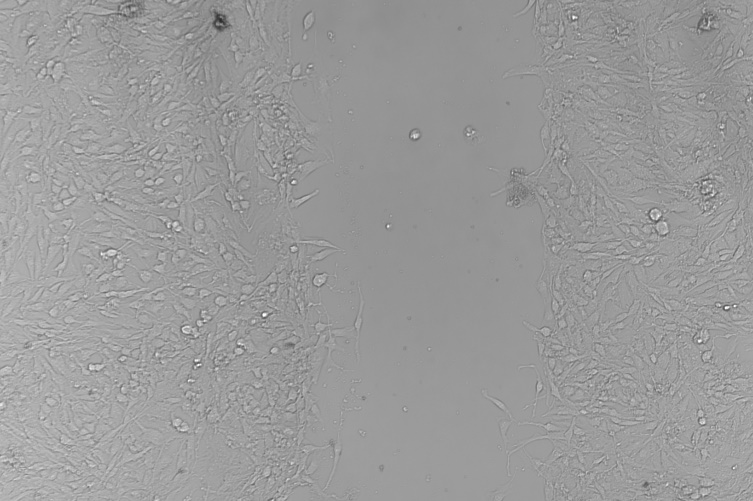


36h:


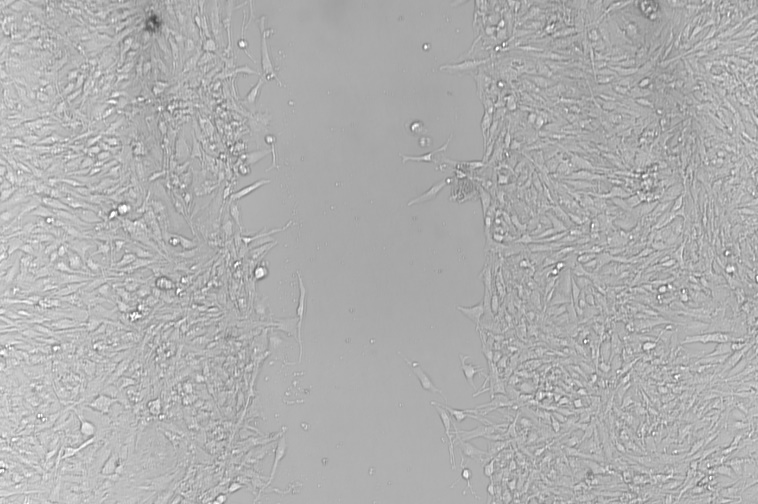


48h:


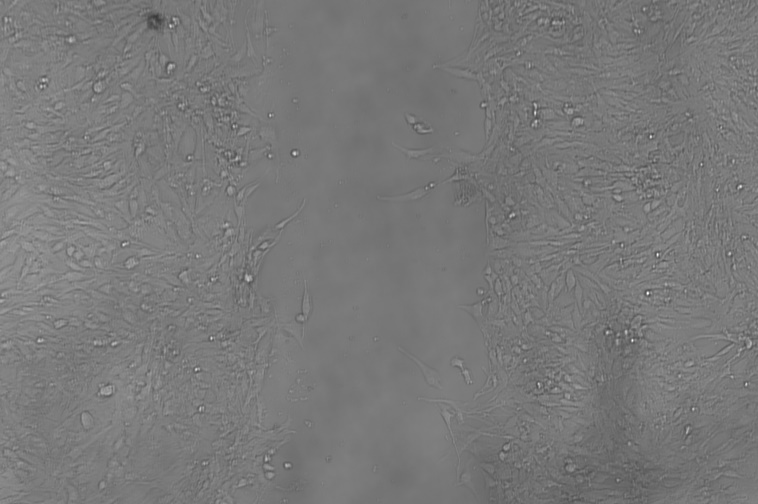


**4. C_18_H_17_NO_6_ 3μM**

0h:


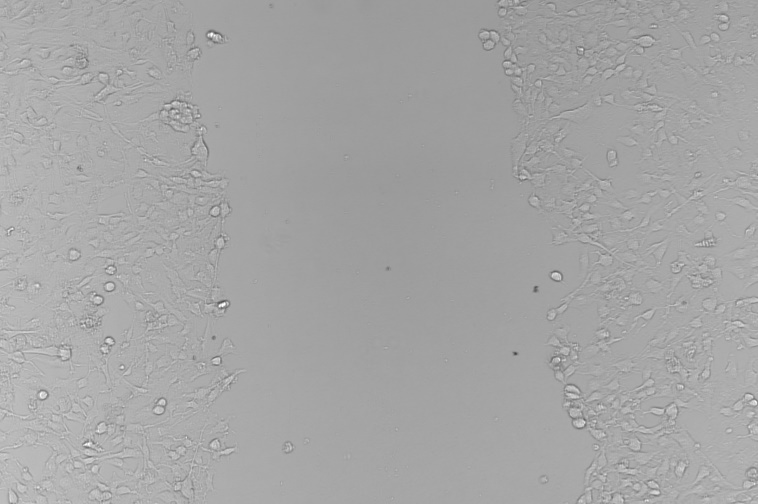


12h:


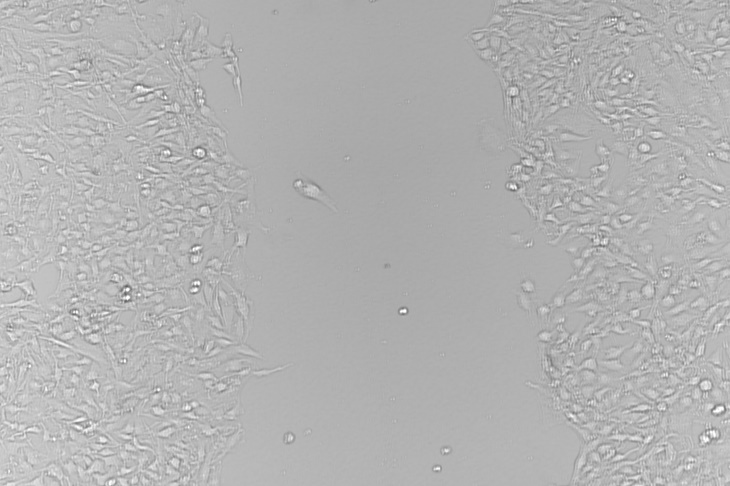


24h:


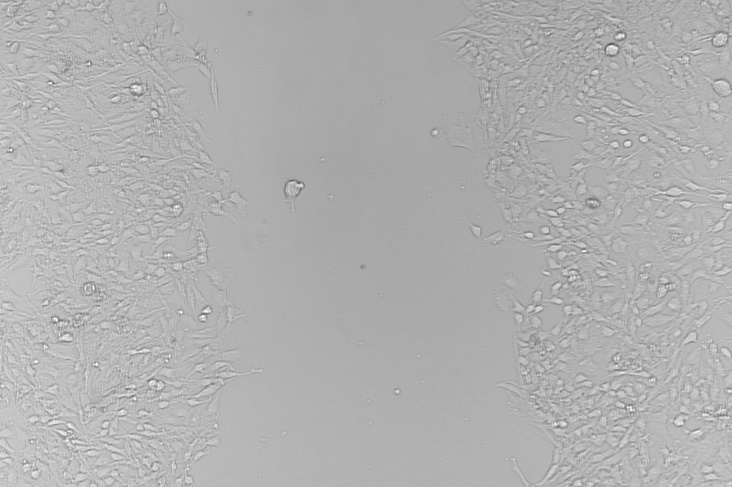


36h:


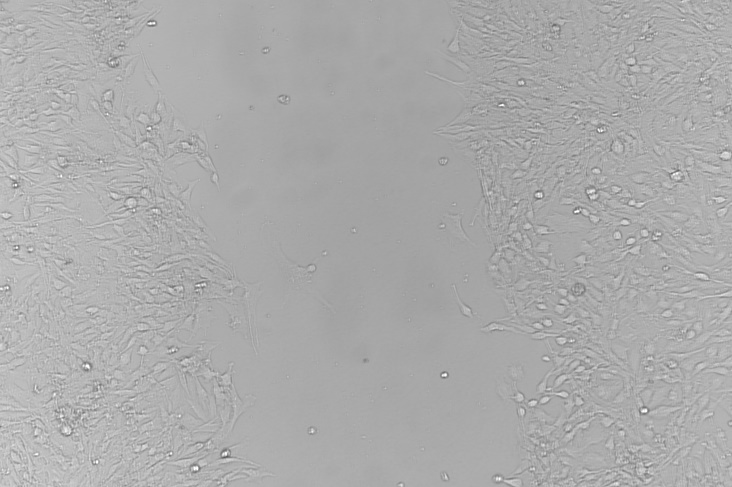


48h:


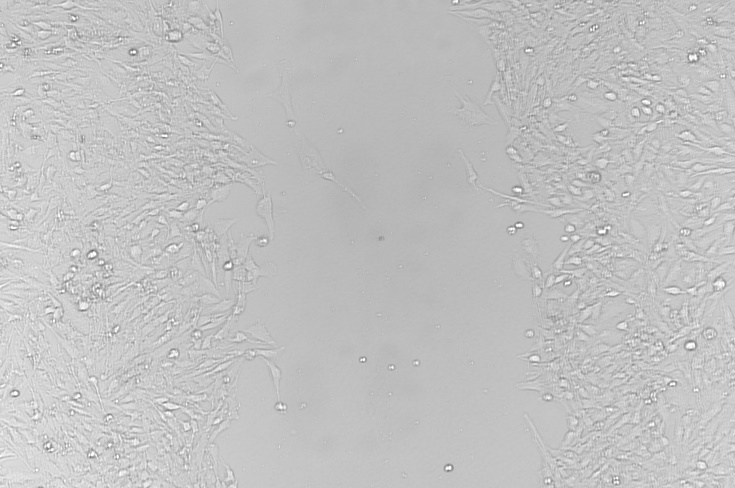


**5. C_18_H_17_NO_6_ 5μM**

0h:


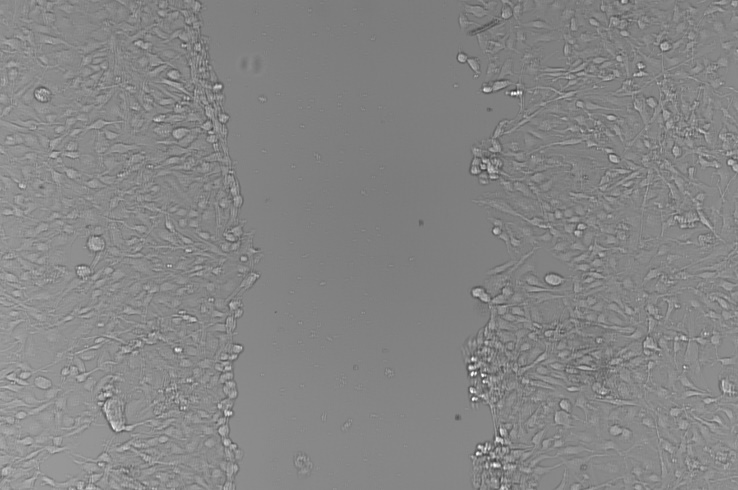


12h:


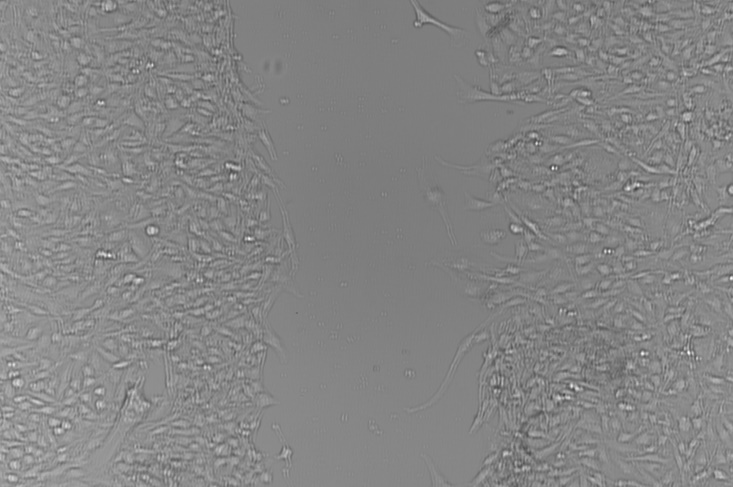


24h:


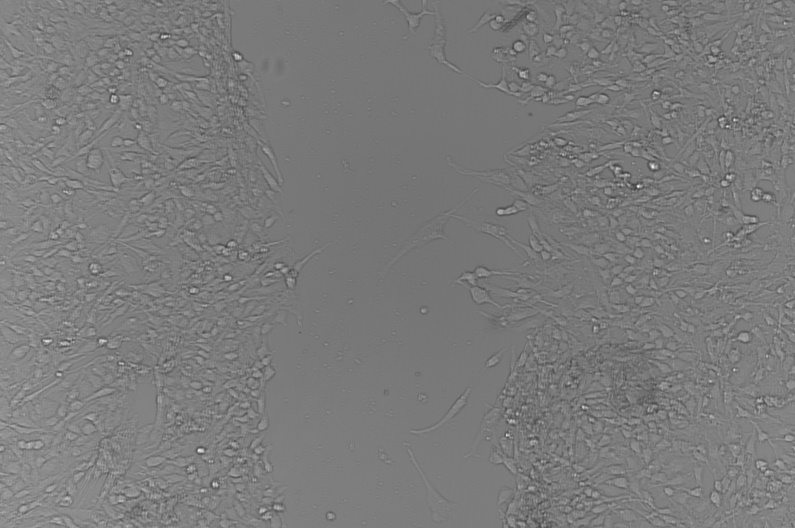


36h:


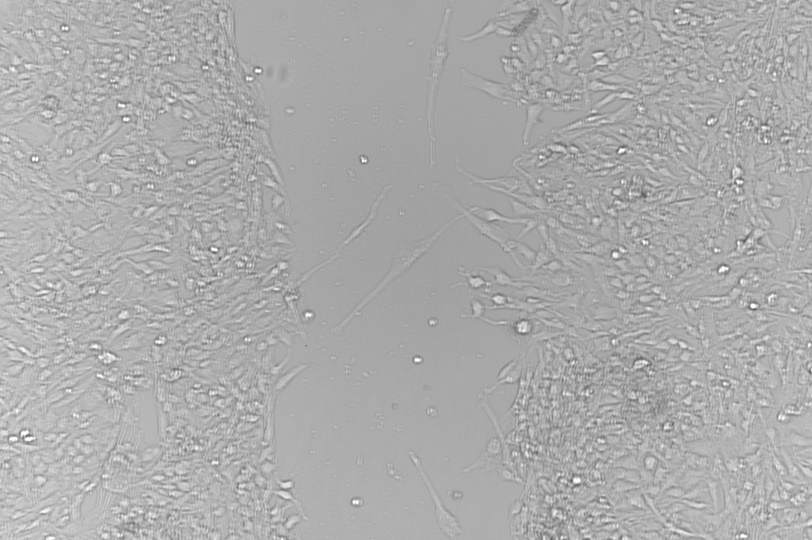


48h:


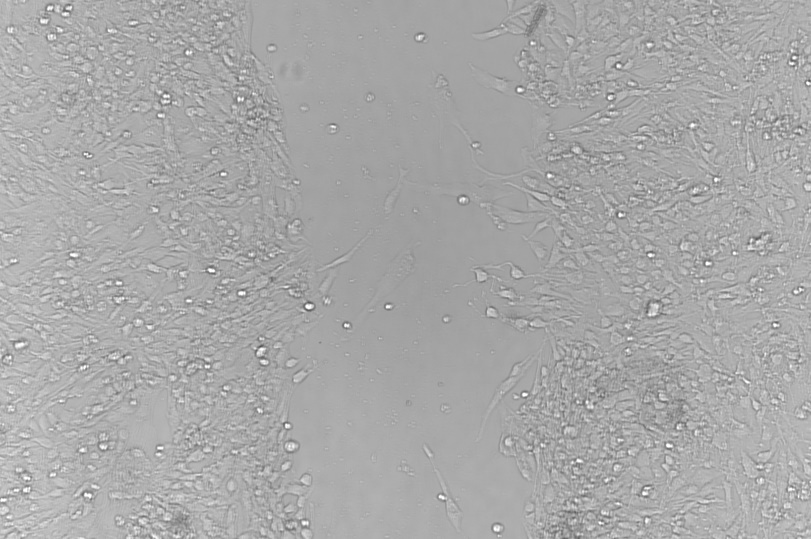


**6. SCU 200μM**

0h:


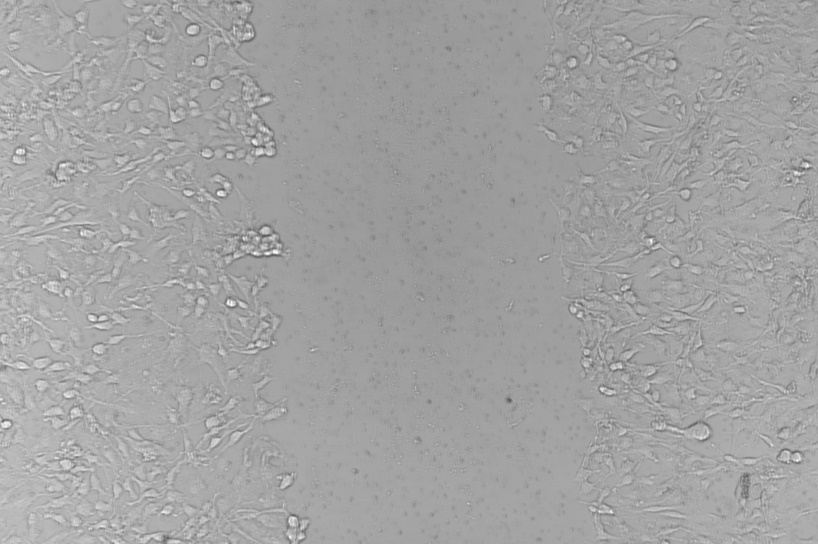


12h:


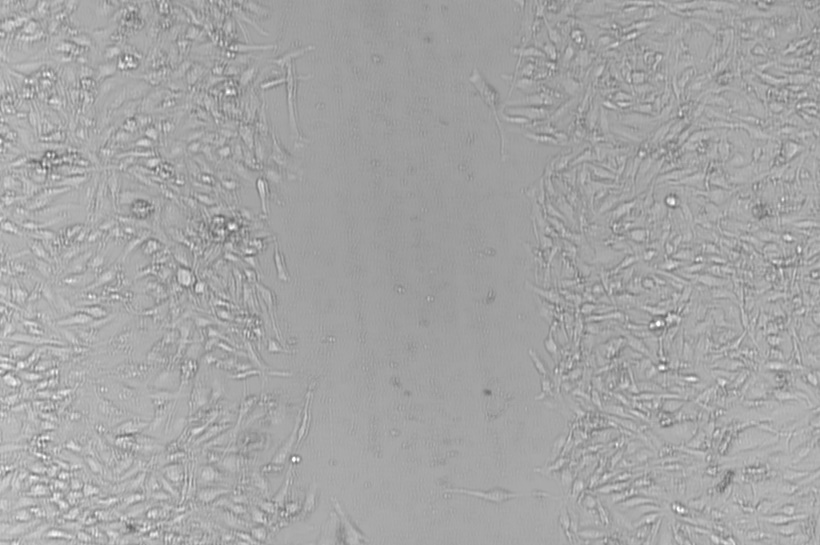


24h:


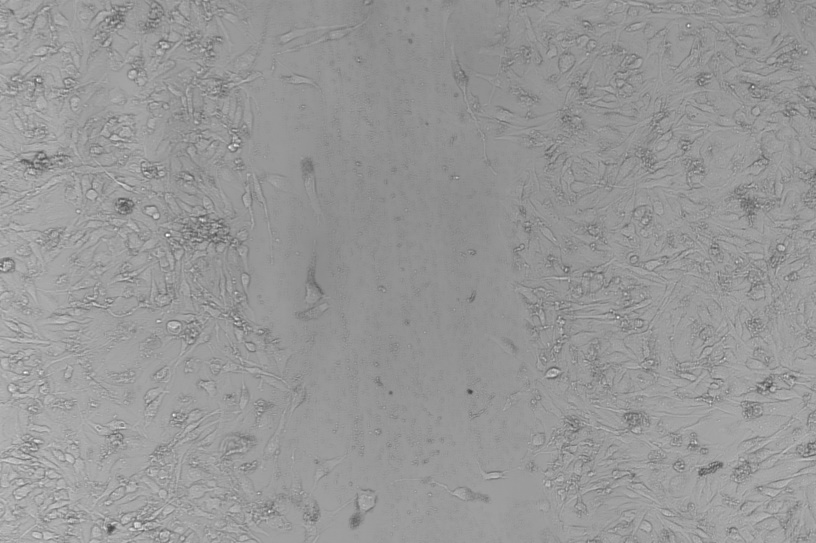


36h:


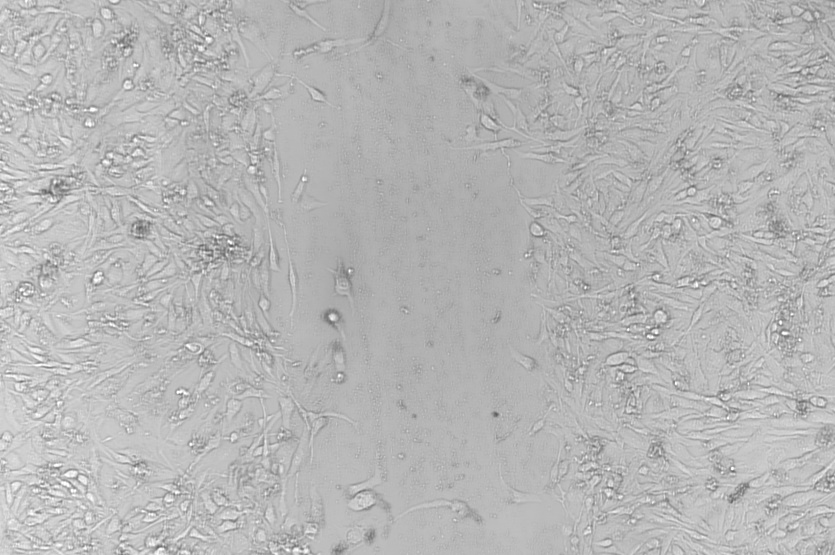


48h:


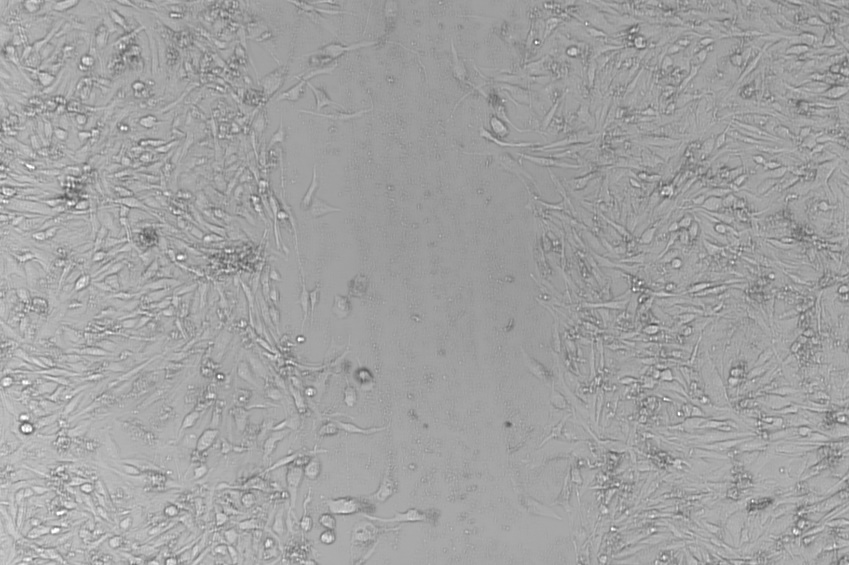


**7. C_18_H_17_NO_6_ 1μM+SCU 200μM**

0h:


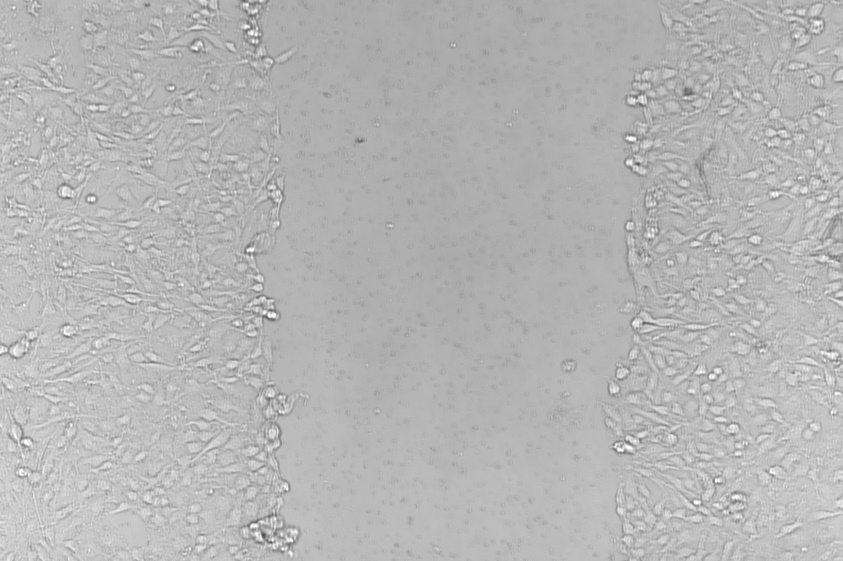


12h:


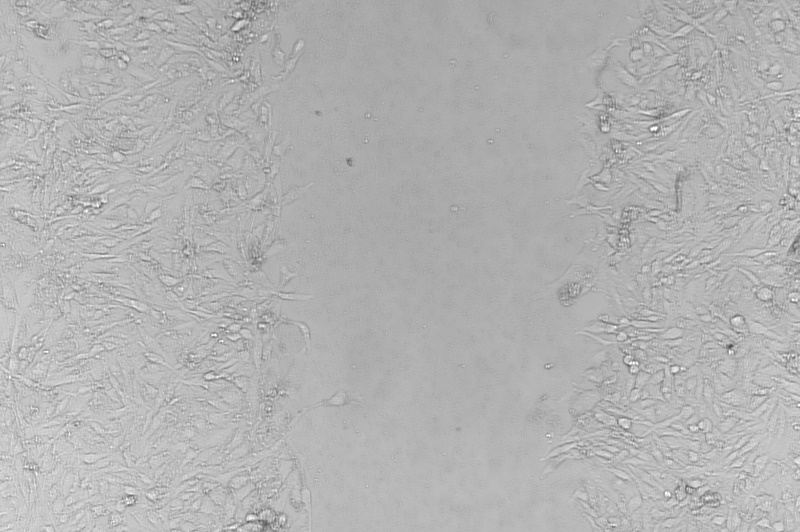


24h:


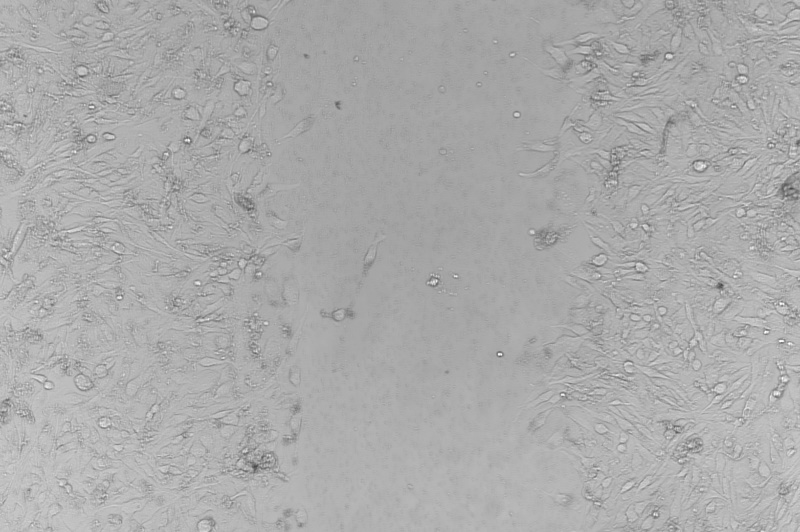


36h:


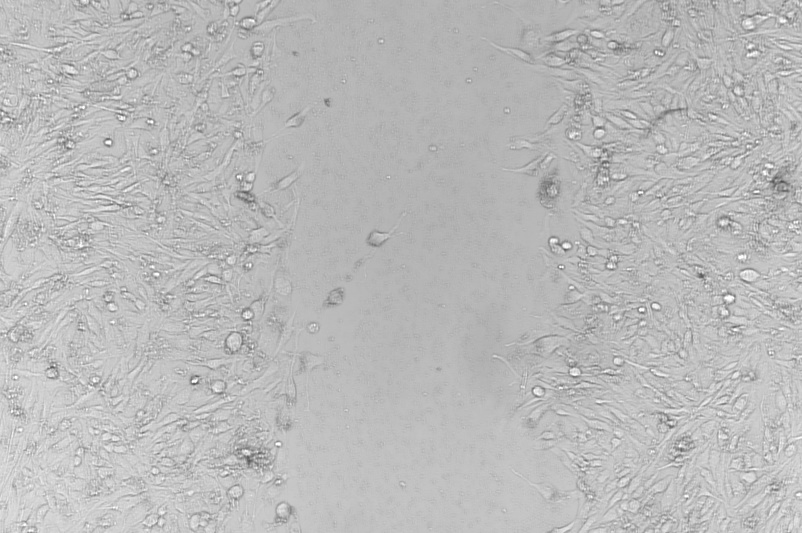


48h:


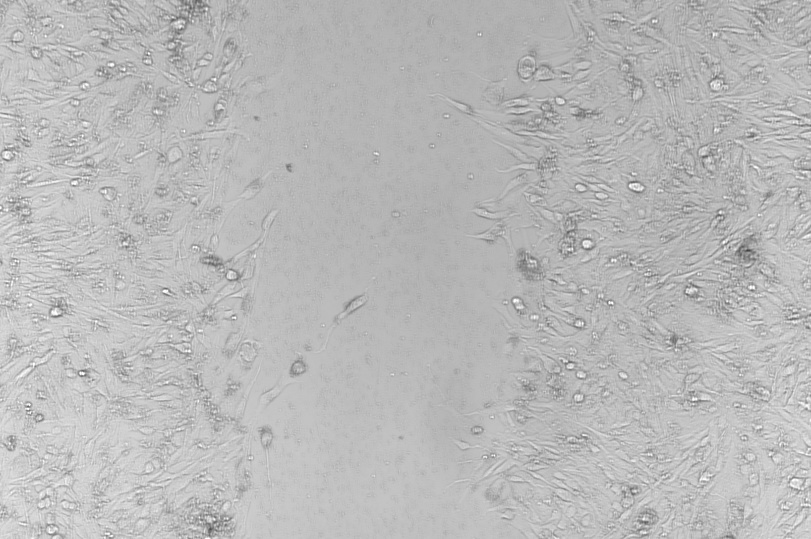


**8. C_18_H_17_NO_6_ 3μM+SCU 200μM**

0h:


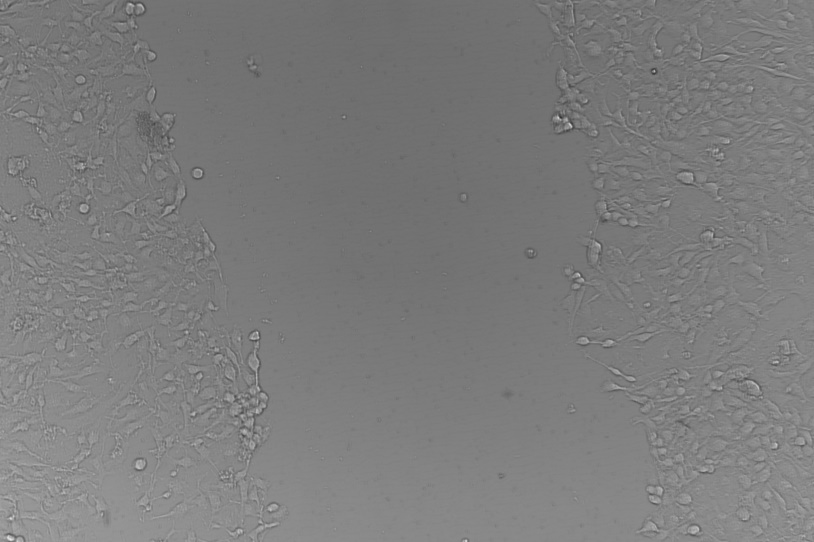


12h:


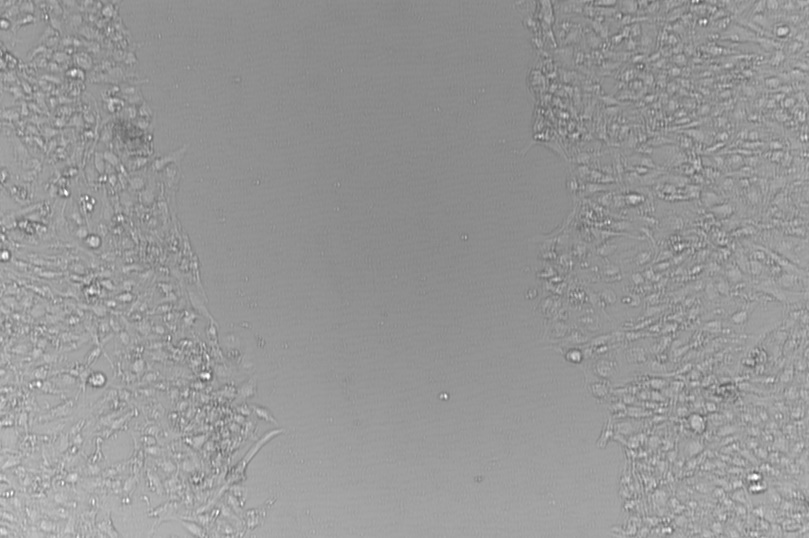


24h:


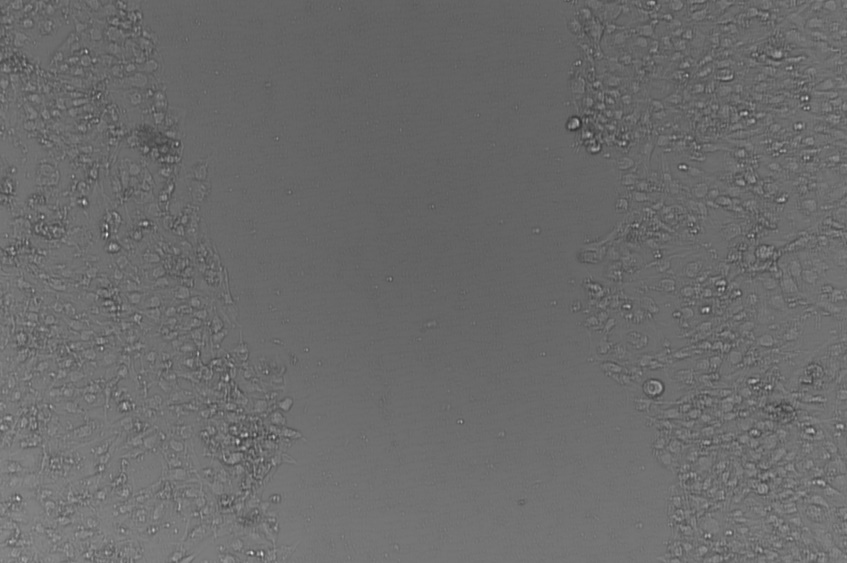


36h:


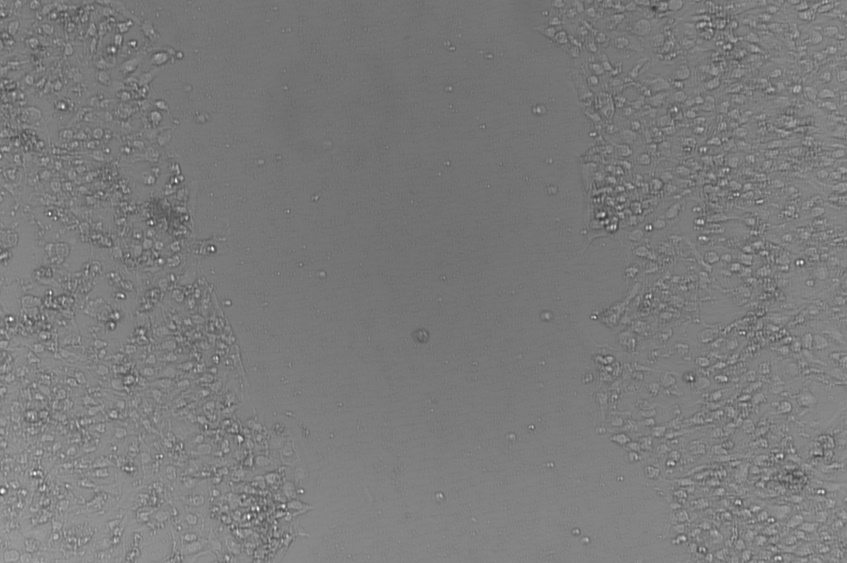


48h:

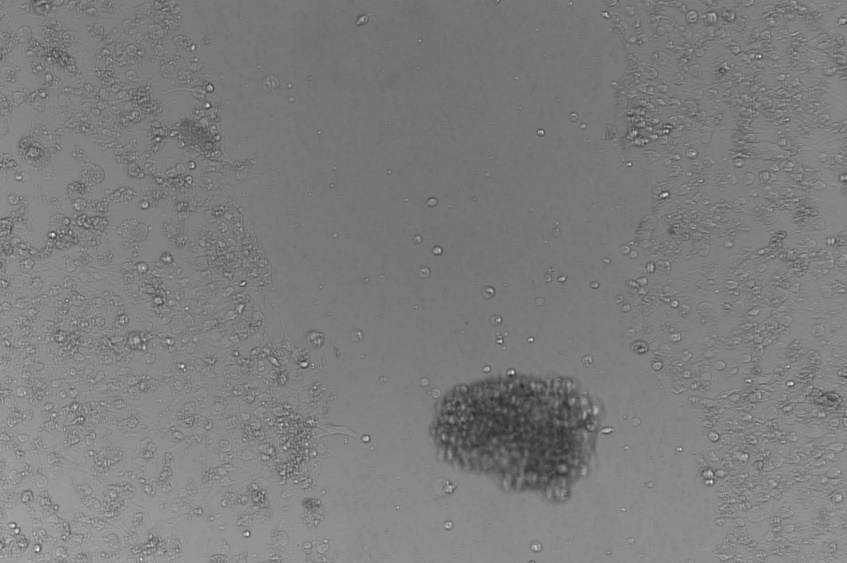


**9. C_18_H_17_NO_6_ 5μM+SCU 200μM**

0h:


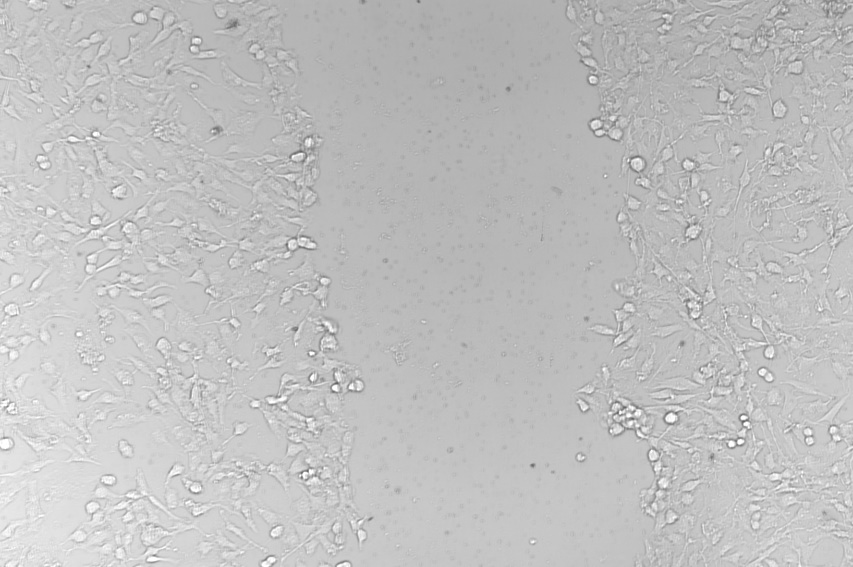


12h:


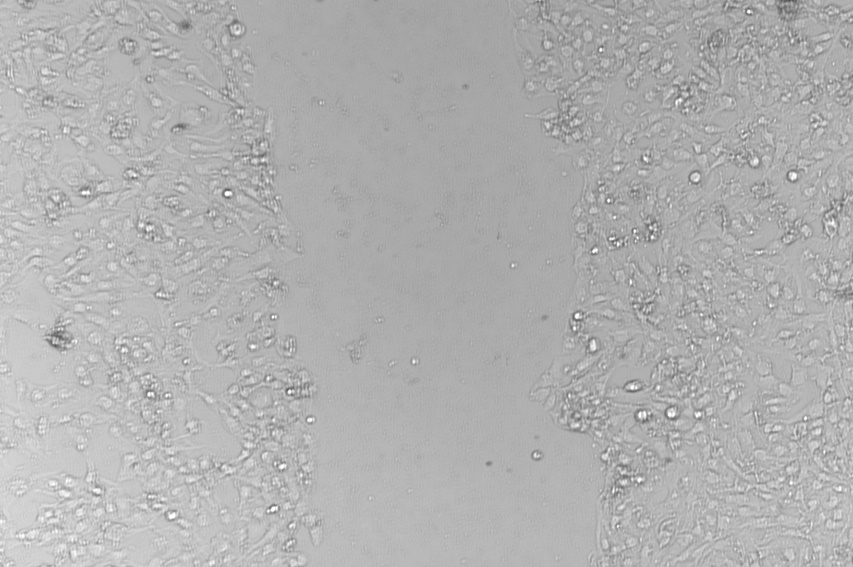


24h:


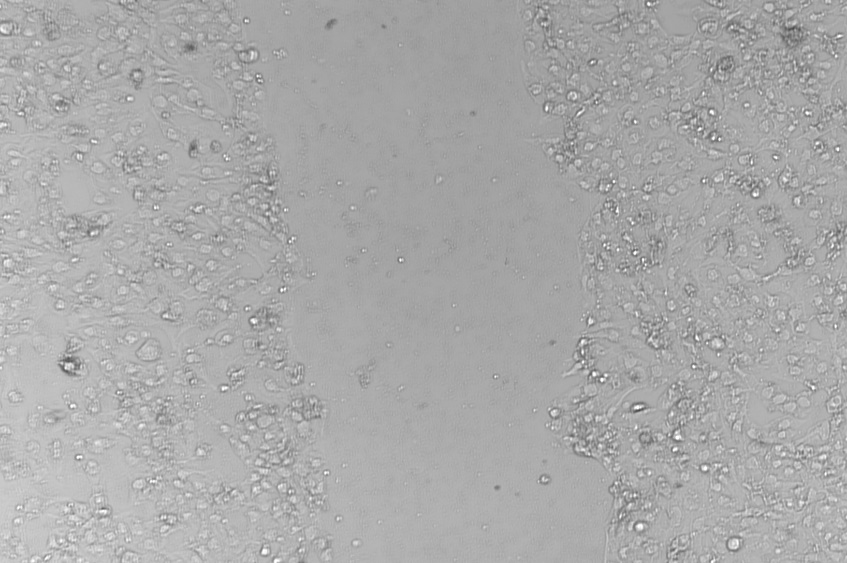


36h:


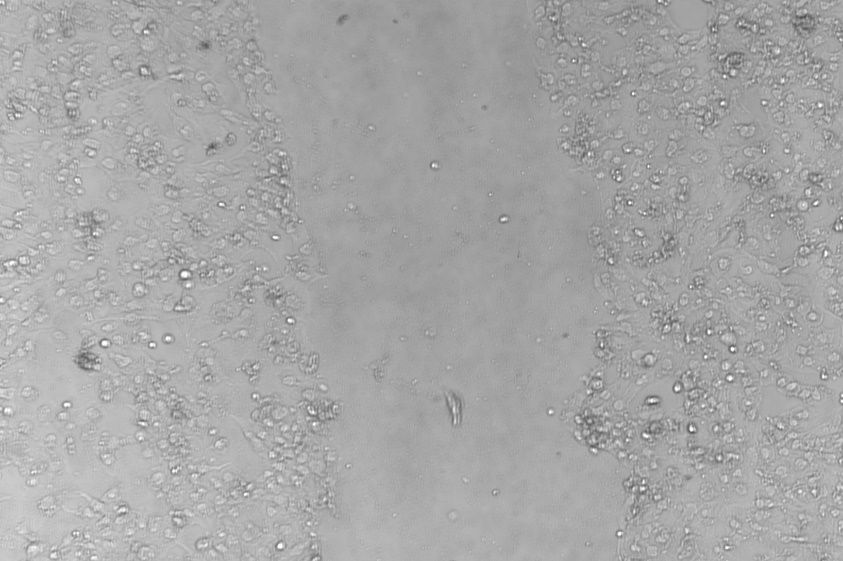


48h:


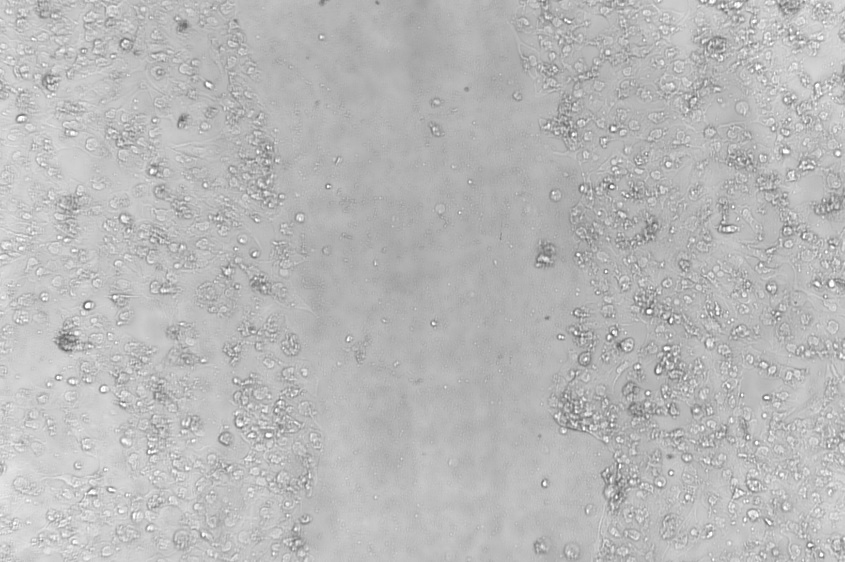

Supplement: Supplementary Materials — Supplementary Data 1: (a) the inhibition rate of C18H17NO6 on glioma cells by CCK8 test; (b) the inhibition rate of Scutellarin on glioma cells by CCK8 test. Supplementary Data 2: (a) the cell viability of glioma cells intervened by C18H17NO6 and its combination with Scutellarin for 24h with cck8 test, (b) the cell viability of glioma cells intervened by C18H17NO6 and its combination with Scutellarin for 48h with cck8 test, and (c) the cell viability of glioma cells intervened by C18H17NO6 and its combination with Scutellarin for 72h with cck8 test. Supplementary Data 3: the clone count of LN229 cell intervened by C18H17NO6 and its combination with Scutellarin for 48h. Supplementary Data 4: (a) the effect of C18H17NO6 and its combination with Scutellarin on the proliferation of U251-the figures of EdU incorporation assay, (b) the effect of C18H17NO6 and its combination with Scutellarin on the proliferation of LN229-the figures of EdU incorporation assay, and (c) the effect of C18H17NO6 and its combination with Scutellarin on the proliferation rate of glioma cells by EdU incorporation assay. Supplementary Data 5: the effect of C18H17NO6 and its combination with Scutellarin on the cell cycle of glioma cells by flow cytometry analysis. Supplementary Data 6: (a) the effect of C18H17NO6 and its combination with Scutellarin on the apoptosis of U251-the figures of TUNEL assay, (b) the effect of C18H17NO6 and its combination with Scutellarin on the apoptosis of LN229-the figures of TUNEL assay, and (c) the effect of C18H17NO6 and its combination with Scutellarin on the apoptosis rate of glioma cells by TUNEL assay. Supplementary Data 7: the effect of C18H17NO6 and its combination with Scutellarin on the apoptosis rate of glioma cells by flow cytometry analysis Supplementary Data 8: (a) the effect of C18H17NO6 and its combination with Scutellarin on the lateral transferred ability of U251-the figures of wound healing assay; (b) the effect of C18H17NO6 and its combination wit [file 6821219.f1.zip › Supplementary data 8/a. U251-the figures of Wound healing assay.docx]
